# Transgenerational Stress Memory Is Not a General Response in Arabidopsis

**DOI:** 10.1371/journal.pone.0005202

**Published:** 2009-04-21

**Authors:** Ales Pecinka, Marisa Rosa, Adam Schikora, Marc Berlinger, Heribert Hirt, Christian Luschnig, Ortrun Mittelsten Scheid

**Affiliations:** 1 Gregor Mendel Institute of Molecular Plant Biology (GMI), Austrian Academy of Sciences, Vienna, Austria; 2 INRA – URGV, Plant Genomics Research Unit, Evry, France; 3 University of Natural Resources and Applied Life Sciences (BOKU), Vienna, Austria; University of Georgia, United States of America

## Abstract

Adverse conditions can trigger DNA damage as well as DNA repair responses in plants. A variety of stress factors are known to stimulate homologous recombination, the most accurate repair pathway, by increasing the concentration of necessary enzymatic components and the frequency of events. This effect has been reported to last into subsequent generations not exposed to the stress. To establish a basis for a genetic analysis of this transgenerational stress memory, a broad range of treatments was tested for quantitative effects on homologous recombination in the progeny. Several Arabidopsis lines, transgenic for well-established recombination traps, were exposed to 10 different physical and chemical stress treatments, and scored for the number of somatic homologous recombination (SHR) events in the treated generation as well as in the two subsequent generations that were not treated. These numbers were related to the expression level of genes involved in homologous recombination and repair. SHR was enhanced after the majority of treatments, confirming previous data and adding new effective stress types, especially interference with chromatin. Compounds that directly modify DNA stimulated SHR to values exceeding previously described induction rates, concomitant with an induction of genes involved in SHR. In spite of the significant stimulation in the stressed generations, the two subsequent non-treated generations only showed a low and stochastic increase in SHR that did not correlate with the degree of stimulation in the parental plants. Transcripts coding for SHR enzymes generally returned to pre-treatment levels in the progeny. Thus, transgenerational effects on SHR frequency are not a general response to abiotic stress in Arabidopsis and may require special conditions.

## Introduction

Living organisms are frequently exposed to limiting or unfavorable environmental conditions. While the majority of animals can escape such conditions by moving or migrating, higher plants, as sessile organisms, only have restricted possibilities of avoiding stress conditions. Plants have, therefore, developed effective survival strategies that are compatible with their sessile lifestyle, which range from short term physiological changes to long term genomic adaptations [1], [2].

Many types of abiotic stress can cause DNA damage, either directly by inducing strand breaks like irradiation or via elevating the level of reactive oxygen species. The late separation of the germ line from somatic tissue in plants also requires stress defense to include potent protection of the genome from accumulation of deleterious mutations, so as to avoid their passage into subsequent generations. Plants have evolved eukaryotic DNA repair systems to very effective networks. There are several major pathways including non-homologous end joining (NHEJ), nucleotide and base excision repair (NER and BER), mismatch repair (MMR) and somatic homologous recombination (SHR) [for reviews see e.g. 3], [4], [5]. The pathways appear to be partially complementary or redundant and form a complex, not yet fully understood system of genome integrity control with different levels of fidelity. While NHEJ is likely the most error-prone pathway that primarily leads to ligation of broken DNA ends without a template, NER/BER/MMR and SHR use the complementary DNA strand or homologous sequences as a master copy, respectively.

Several studies have demonstrated that SHR is one of the general plant responses to stress since many abiotic as well as biotic stress treatments (e.g. UV irradiation, radiomimetic drugs, herbicides, osmotic stress, high temperature) increase the frequency of SHR significantly [6]–[13]. In most experiments, SHR was monitored with transgenic “SHR trap” constructs, consisting of two incomplete but overlapping parts of reporter genes encoding for a selectable or visible marker. The two parts are homologous over at least several hundred base pairs and are arranged in either inverted or direct orientation. Upon each SHR event, a functional version of the transgene is restored and SHR can be monitored in a quantitative manner [9], [14], [15], [for review see e.g. 16]. This allowed mutant screens to identify SHR components as well as systematic tests of drugs and environmental conditions for their effect on SHR as mentioned above.

A tight cell cycle DNA damage checkpoint helps to avoid DNA damage being transmitted to daughter cells [17]. Moreover, DNA damage should be repaired before the genetic material is transmitted to the next generation. Thus, until recently, it was expected that any stress-induced stimulation of the repair processes would diminish together with the trigger. This view was challenged by a study that detected an increased frequency of SHR not only in the treated generation, but also in subsequent, non-stressed generations [18]. This transgenerational stress memory was described after UV-C irradiation or treatment with the elicitor flg22 that mimicks pathogen attack and thus biotic stress. The transgenerational stress memory could be induced with both treatments in several reporter lines carrying different variants of SHR traps at different genomic positions and in two different Arabidopsis ecotypes. However, the mechanistic basis of the transgenerational stress memory remains unknown. Since it was not genetically coupled to the SHR trap and evident as a dominant trait already in the first post-stress generation, it was suggested that it might have an epigenetic basis [18]. The connection of several protein factors known to exert epigenetic control on the genome (such as BRU1, FAS1 and FAS2) with the SHR pathway [19]–[21] also supported this assumption.

The aim of the experiments described here was to reveal whether transgenerational memory depends on the type of stress and to study the molecular basis of the underlying mechanism. Since published data implied that physical or chemical treatments and pathogen perception induced SHR and changed gene expression in a similar way [13], we restricted our analysis to abiotic stress factors and rather diversified the range of parameters and doses. We performed a screen in which we exposed two Arabidopsis SHR trap reporter lines 11 and 1445 [14], [22] with relatively high recombination rates to ten different abiotic stress treatments at different dosages. We show that SHR is enhanced after the majority of treatments, confirming previous data and adding new effective stress types such as interference with chromatin modifications. The most pronounced stimulation was achieved by treatment with compounds that directly modify or damage DNA. Responses to these drugs can exceed previously achieved values, indicating that the capacity for SHR is not easily exhausted. In spite of the significant stimulation in the stressed generations, we did not generally find increased SHR levels in two subsequent generations obtained by selfing treated plants. The expression of several genes that encode protein factors involved in homologous recombination processes were high in the stress-treated plant material but returned to pre-treatment levels in the next generations, in good correlation with the SHR data. Thus, transgenerational effects on SHR frequency are not a general response of plants to abiotic stress.

## Results

### SHR can be effectively stimulated by many types of abiotic stress

SHR frequencies were scored using SHR trap lines that allow quantitative evaluation of SHR by counting individual recombination events that restore the ß-glucuronidase gene (GUS), visible as blue spots after histological staining [14]. Several SHR trap lines were previously described to exhibit increased recombination frequencies after distinct stress treatments, although with different baselines and to different extents. Based on initial experiments with nine different lines in two different ecotypes and three stress treatments, we selected two SHR marker lines 11 and 1445 [14], [22] for detailed analysis. These lines have the SHR trap inserted in gene-rich (euchromatic) regions on the bottom arm of chromosome 2. Both lines were exposed to an extended variety of abiotic stress types (Table 1). These include conditions previously shown to stimulate SHR in these and/or other SHR trap lines: salt, heat, cold, radiomimetic (bleocin) or oxidative (paraquat) drugs, UV-B and UV-C [6], [8]–[11], [13], [18], [23]–[26]. To test for a potential role of epigenetic factors in the control of SHR [19], [21], we further applied drugs previously not tested and affecting either DNA methylation [zebularine; 27] or histone modifications [trichostatin A, sodium butyrate; 28]. For most of the treatments, we applied different doses and determined the SHR frequency shortly thereafter in the stressed plants, termed S0 (S for stressed), thereby selecting maximal induction of SHR over the mock treatments. All data are expressed as relative values against the mock-treated plants of the same generation grown in parallel. All absolute values are listed in the supplementary tables S1, S2, S3, S4, S5, S6, S7, S8, S9, S10, S11, S12, S13, S14, S15, S16, S17.

**Table 1:** Frequency of homologous recombination in stressed S0 generation.

Type of stress	Dose	Fold change	
		Line 11	Line 1445
		Generation S0	Generation S0
Salt ^Table S1^	100 mM NaCl	3.0***	1.6
Osmotic ^Table S2^	Mannitol	2.1***	1.0
Freezing ^Table S3^	−4°C	2.2***	0.7
Heat ^Table S4^	37°C	2.8***	3.4*
Radiomimetic ^Table S5^ ^, ^ ^S6^ ^, ^ ^S7^	10 ng/ml Bleocin	2.9***	15.0***
	20 ng/ml Bleocin	2.1**	21.0***
	50 ng/ml Bleocin	3.0***	22.6***
	100 ng/ml Bleocin	2.7***	52.4***
	200 ng/ml Bleocin	4.3***	54.8***
	400 ng/ml Bleocin	3.7***	113.7***
Oxidative ^Table S8^	0.1 µM Paraquat	2.3***	0.8
	0.25 µM Paraquat	2.6***	0.7
	0.5 µM Paraquat^5,14^	1.2	0.2**
	1 µM Paraquat	1.2	0.2**
UV-B ^Table S10^	3.1 kJ/m^2^/day - 8 days	n.d.	4.9**
	4.7 kJ/m^2^/day - 8 days	n.d.	7.3***
	6.3 kJ/m^2^/day - 8 days	n.d.	7.4***
UV-C ^Table S11^	1×750 J/m^2^	1.1	4.8***
	2×1500 J/m^2^	0.6*	6.4***
	1×3000 J/m^2^	0.8	7.7***
DNA demethylation ^Table S12^ ^, ^ ^S13^ ^, ^ ^S14^	20 µM Zebularine	6.7***	75.9***
	40 µM Zebularine	12.7***	143.3***
	80 µM Zebularine	7.4***	152.2***
Histone hyper-acetylation ^Tables S15^ ^, ^ ^S16^	1 µg/ml Trichostatin-A^10^	0.4***	2.6*
	0.4 mM Na-butyrate^11^	0.6*	4.7***

Fisher's exact test: *P<*0.05*, **0.001<*P*<*0.01*, ***P<*0.001*.

n.d. = not done.

Superscript numbers indicate supplementary tables with raw data.

Line 11 responded with a highly significant increase in SHR (*P*<*0.01*) to the majority of treatments, confirming all earlier reports on the effect of salinity and cold stress [11], [26] including the related stress of increased osmolarity. Unchanged or even decreased numbers of SHR events were observed after higher doses of paraquat, all doses of UV-C, trichostatin A and sodium butyrate (Table 1). Line 1445 seems to be more specific in its response since it is highly stimulated by zebularine, sodium butyrate, bleocin and UV, less by heat and trichostatin A, and shows no significant increase after paraquat, salt and freezing stress (Table 1).

Several stresses seem to stimulate SHR in Arabidopsis more universally than others. We observed a significant increase with both tested lines exposed to heat, bleocin and zebularine. Heat increased SHR 2.8- and 3.4-fold in lines 11 and 1445, respectively, and also had a significant effect on SHR in lines 651 and IC9 (Table S4). The mechanism of SHR stimulation by heat is currently unknown. Bleocin caused an increase by 2.1- to 4.3-fold and 15- to 113-fold in lines 11 and 1445, respectively. In the presence of metal ions, bleocin forms a pseudoenzyme that reacts with oxygen and produces superoxide and free hydroxide radicals, which then induce DNA strand breaks [29]. Zebularine increased SHR up to 6.7- and 152-fold in lines 11 and 1445, respectively, to our knowledge the highest increase in SHR reported so far. Zebularine is a cytosine analog that, upon incorporation into DNA, covalently binds DNA methyltransferases and leads to global genome DNA demethylation and transcriptional reactivation of epigenetically silent genes and transgenes in Arabidopsis [27].

Another class of chromatin-affecting drugs was represented in this study by sodium butyrate and trichostatin A. Both chemicals inhibit class I and II histone deacetylases (HDAC) and lead to histone hyperacetylation which can result in release of epigenetic gene silencing and/or stimulated transcription [30], [31]. Both compounds increased the SHR frequency in line 1445 significantly (Table 1), however, not at all to the levels achieved by the radiomimetic drug and the methylation inhibitor. In line 11, the application of both drugs seemed to have a rather suppressive effect on SHR (Table 1).

Paraquat is widely used to induce oxidative stress. It produces superoxide radicals that can damage cellular membranes as well as DNA [32], [33]. While lower concentrations of paraquat stimulated SHR only in line 11, higher concentrations of this drug suppress SHR in both tested lines (Table 1). This is very likely due to the deleterious and toxic effect of paraquat treatment: more than 0.25 µM reduced plant growth and development even after removal of the drug (data not shown).

Although not identical in terms of perception and damage [34], both types of UV irradiation applied here gave a similar and significant increase in SHR frequency for line 1445 (Table 1). This indicates that the repeated application of UV-B with a longer wavelength spectrum over several days caused a comparable effect to the single dose of the more energy-rich UV-C. The lack of a response in line 11 to a single dose of UV-C was rather surprising since line 11 was reported to respond well to this type of stress [18], [23]. Non-responsiveness was confirmed in several independent experiments and with an extended range of UV doses (Table S17). Nevertheless, marker genes of the homologous recombination repair pathway (*RAD*51 and *MIM*) were significantly induced (Figure S1).

In summary, we determined specific doses of various physical and chemical abiotic stress types that significantly increase the SHR frequency in the treated S0 generation. These comprise conditions that were previously shown to trigger SHR, as well as new treatments with even stronger SHR induction. On this basis, we tested for the presence of transgenerational stress memory by scoring SHR in the subsequent two generations.

### Significant increase of SHR in S1 and S2 generations is rare and stochastic

Seeds of lines 11 and 1445 obtained from self-pollinated S0 populations with increased SHR frequencies were germinated and grown for two generations (S1 and S2) without stress treatment. In each generation, the number of SHR events per plant was scored and compared to populations from mock-treated plants grown under the same standard conditions.

Among 15 progeny populations obtained by selfing from line 11 S0 plants with significantly enhanced SHR after stress treatments, most did not show any significant increase of SHR in the S1 generation (Table 2). The only exceptions were the S1 progeny of S0 plants stressed with 0.1 µM and 0.25 µM paraquat which showed a 6.4- and 5.7-fold increase compared to the corresponding S1 mock (both *P*<*0.001*), respectively. This increase in S1 was even stronger than that of the S0 generation – indeed suggesting a transgenerational stress memory effect after paraquat stress. However, the subsequent S2 generation showed no or only a very small and non-significant increase in SHR events (Table 2 and Table S8). This would indicate a more rapid loss of the stress memory after paraquat than the long-lasting effect (at least 4 generations) previously described after UV-C treatment [18]. In addition, the effect may not always reach the progeny. A repetition of line 11 treatment with 0.1 µM paraquat under conditions as close as possible to the first set of experiments yielded a weaker but still significant 2.3-fold SHR increase (*P* = *0.022*) in S0. From this S0 population, five paraquat-stressed plants (and two plants from the mock-treated population) were grown to maturity and seeds were harvested separately per plant, to determine the level of variation between individual plants. Mock S1 plants showed only little variation in the frequency of SHR (5.9 and 6.1 spots/plant). The variation was larger among progeny of paraquat-stressed plants (3.5, 6.9, 5.7, 6.3 and 6.6 spots/plant). However, none of these values was significantly higher than that in the mock plants (Table S9). Thus, paraquat may possibly lead to transgenerational stress memory. However, the induction seems to occur in a rather stochastic manner which makes it difficult to study the underlying mechanism in a controlled way. The only other increased SHR frequency in a group of progeny that was scored as relevant using statistical tests came from plants treated with 40 µM zebularine (Table 2). In this case, the increase was much less pronounced than in S0 and was limited to one concentration of the drug and again to one generation only. No increase above insignificant levels was seen in any S2 population. Some stress treatments even seemed to have a suppressive effect on SHR in the S1 and S2 progeny (Table 2).

**Table 2:** Frequency of homologous recombination in progeny of stressed plants of line 11.

Type of stress	Dose	Fold change		
		Generation S0	Generation S1	Generation S2
Salt ^Table S1^	100 mM NaCl	3.0***	1.0	0.8***
Osmotic ^Table S2^	Mannitol	2.1***	0.6***	0.5***
Freezing ^Table S3^	−4°C	2.2***	1.3	0.9
Heat ^Table S4^	37°C	2.8***	0.5*	1.2
Radiomimetic ^Table S5^ ^, ^ ^S6^ ^, ^ ^S7^	10 ng/ml Bleocin	2.9***	1.4	1.7
	20 ng/ml Bleocin	2.1**	1.1	0.8
	50 ng/ml Bleocin	3.0***	1.2	0.5
	100 ng/ml Bleocin	2.7***	1.1	0.9
	200 ng/ml Bleocin	4.3***	1.1	0.8
	400 ng/ml Bleocin	3.7***	1.7	1.2
Oxidative ^Table S8^ ^, ^ ^S9^	0.1 µM Paraquat	2.3***	6.4***	1.0
	0.25 µM Paraquat	2.6***	5.7***	1.4
DNA demethylation ^Table S12^ ^, ^ ^S13^ ^, ^ ^S14^	20 µM Zebularine	6.7***	1.2	1.7***
	40 µM Zebularine	12.7***	1.9***	0.9
	80 µM Zebularine	7.4***	1.2	1.1

Fisher's exact test: *P<*0.05*, **0.001<*P*<*0.01*, ***P<*0.001*.

Superscript numbers indicate supplementary tables with raw data.

For 18 populations obtained from the different treatments of line 1445, we found only one population (50 ng/ml bleocin) showing an increase in S1, and again none among the S2 generations (Table 3). However, since the difference is only slightly significant (*P* = *0.020*), and lower as well as higher doses did not have such an effect – we consider this difference to rather be due to experimental variation. It should be emphasized that although stimulation of SHR in line 1445 was much more pronounced and reached higher absolute numbers (Table S5 and S12), even populations with an over 100-fold increased SHR frequency in S0 (400 ng/ml bleocin, and 40 and 80 µM zebularine) did not show a significant increase in S1 progeny compared to mock-treated plants. Therefore, the degree of initial response does not seem to determine the level of transgenerational effects.

**Table 3:** Frequency of homologous recombination in progeny of stressed plants of line 1445.

Type of stress	Dose	Fold change		
		Generation S0	Generation S1	Generation S2
Heat ^Table S4^	37°C	3.4*	1.0	0.9
Radiomimetic ^Table S5^ ^, ^ ^S6^ ^, ^ ^S7^	10 ng/ml Bleocin	15.0***	1.7	1.0
	20 ng/ml Bleocin	21.0***	1.4	0.8
	50 ng/ml Bleocin	22.6***	2.3*	0.9
	100 ng/ml Bleocin	52.4***	1.9	1.4
	200 ng/ml Bleocin	54.8***	1.8	0.8
	400 ng/ml Bleocin	113.7***	1.0	0.8
UV-B ^Table S10^	3.1 kJ/m^2^/day - 8 days	4.9***	1.0	2.5
	4.7 kJ/m^2^/day - 8 days	7.3***	1.6	3.3
	6.3 kJ/m^2^/day - 8 days	7.4***	1.5	3.3
UV-C ^Table S11^	1×750 J/m^2^	4.8***	0.8	0.6**
	2×1500 J/m^2^	6.4***	0.6*	n.d.
	1×3000 J/m^2^	7.7***	0.7*	0.4***
DNA demethylation ^Table S12^ ^, ^ ^S13^ ^, ^ ^S14^	20 µM Zebularine	75.9***	1.1	0,8
	40 µM Zebularine	143.3***	1.0	1,1
	80 µM Zebularine	152.2***	0.7	0.7**
Histone hyper-acetylation ^Tables S15^ ^, ^ ^S16^	1 µg/ml Trichostatin-A^10^	2.6*	1.2	1.0
	0.4 mM Na-butyrate^11^	4.7***	0.7	0.8

Fisher's exact test: *P<*0.05*, **0.001<*P*<*0.01*, ***P<*0.001*.

n.d. = not done.

Superscript numbers indicate supplementary tables with raw data.

Additional data, with a limited selection of stress types, confirmed strong S0 effects but a lack of S1 and S2 effects for recombination trap lines 651 and IC9 as well (Text S1 and Tables S4, S12, S13, S14, S17).

In summary, the few cases of significantly increased SHR within all S1 and S2 populations of four SHR trap lines appeared stochastic rather than strictly related to the applied treatments, indicating a strong influence of additional parameters beside possible transgenerational stress memory. Therefore, this phenomenon does not seem to be a general response of Arabidopsis to SHR stimulation by abiotic stress.

### Genes involved in SHR become up-regulated upon stress in S0 but not in S1 and S2

**Figure 1:**
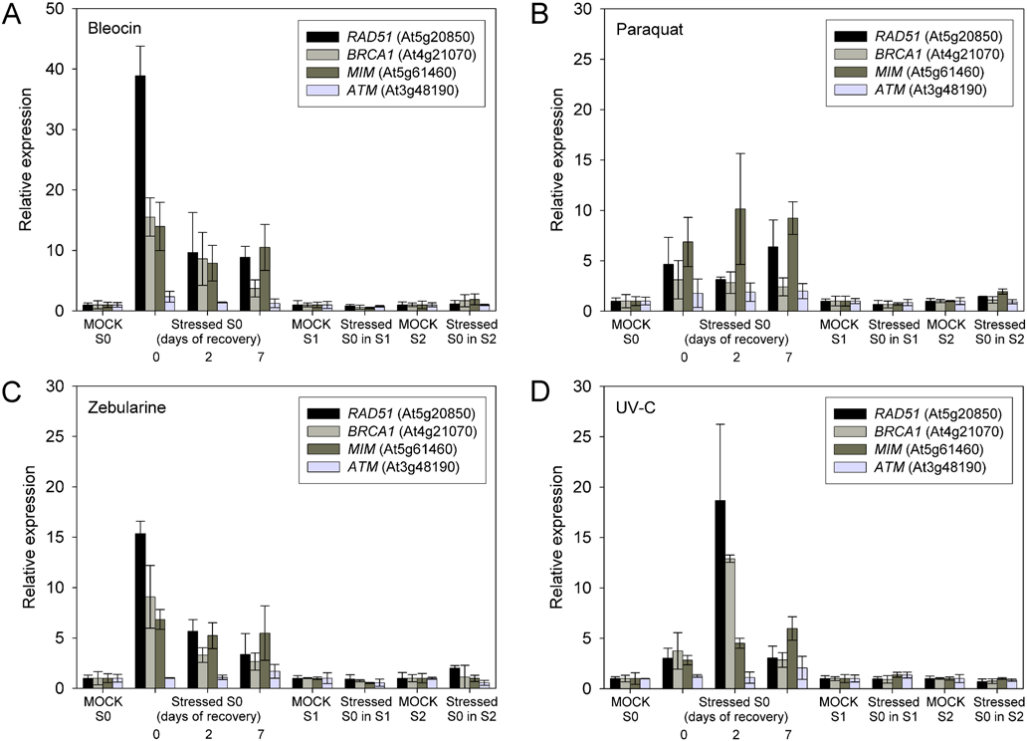
Expression of genes involved in homologous recombination in treated S0 and untreated S1 and S2 generations. Relative expression after bleocin (A), paraquat (B), zebularine (C) and UV-C (D) treatment was measured by real-time PCR (with *UBC28* as a reference gene not influenced by any treatment) as the amount of transcript in stressed S0 and non-stressed S1 and S2 progeny plants of line 1445 and normalized to the amount of transcript in corresponding mock-treated S0, S1 and S2 generations, respectively. *RAD51*, *BRCA1* and *MIM* were strongly up-regulated in S0 by bleocin, zebularine and UV-C and, to a weaker but significant degree, by paraquat. Up-regulation after UV-C is apparent only at the second time point since plant material was harvested immediately after irradiation. *ATM* showed only minor and mostly non-significant changes in expression after stress. None of the tested genes showed an increased amount of transcript in the non-stressed S1 and S2 progeny.

To analyze possible transgenerational stress memory independently of the transgenic SHR trap loci, we measured expression of several genes known to be involved in SHR such as *RAD*51, *BRCA*1, *MIM* and *ATM* [35]–[40]. Expression was monitored by real-time PCR in the S0 generation immediately after stress treatment as well as after two and seven days of recovery. In addition, we compared transcript levels to those of corresponding mock plants in the non-stressed S1 and S2 generations as well. We used line 1445 after the two effective treatments with bleocin (100 ng/ml) and zebularine (40 µM), as well as paraquat (0.1 µM) which caused increased SHR in at least one of the S1 generations, and with UV-C (3000 J/m^2^) previously reported to cause transgenerational stress memory [18]. In the treated generation, *RAD51*, *BRCA1* and *MIM* were strongly upregulated by bleocin and zebularine (Figures 1A–C) immediately after treatment and remained significantly above pre-treatment levels even one week after recovery. Paraquat also induced higher transcript levels of the repair genes, although the effect was less pronounced and thereby in agreement with the weaker increase of SHR events. UV-C treatment also led to a strong induction of homologous recombination genes with the highest expression after 2 days of recovery. The weaker expression at the zero time point likely reflects the fact that tissue for RNA extraction was sampled immediately after the irradiation, not leaving enough time for full activation of the homologous recombination pathway. The checkpoint kinase *ATM* that labels the sites of DNA strand breaks via phosphorylation of histone H2A.X [35] that can be processed via NHEJ or HR pathways, was not significantly induced upon the treatments in S0 (Figure 1D). This is in agreement with published data for bleocin treatment (http://www.genevestigator.ethz.ch) and may reflect a more tissue- or stage-specific role of ATM [41].

As the up-regulation of three SHR genes upon stress in S0 corresponded well with an increased number of SHR events monitored by the trap constructs, the correlation also holds true for non-stressed S1 and S2 populations originating from the stressed S0 plants. None of the marker genes with increased transcript levels in S0 showed significant expression changes in S1 or S2, compared to the corresponding mock-treated plants (Figures 1A–D). This is in agreement with the non-elevated expression levels of marker genes mentioned by Molinier et al. (2006). Thus, the increased transcript levels of SHR gene are not maintained into subsequent generations, a result congruent with the return to baseline levels of SHR frequency.

## Discussion

### Responses of recombination lines

Among the different DNA repair mechanisms, homologous recombination is certainly extremely important for long-term genome fitness and stability. It reconstitutes damaged single copy sequences with high fidelity. However, when acting on repeats, it can also contribute to genome rearrangements and genetic diversity. It is plausible that such a pathway could be included in general plant defense strategies in response to adverse conditions, and an increased activity of the SHR pathway was detected even after stresses that are not known to cause direct DNA damage but act more indirectly, e.g. sodium chloride, freezing, or pathogen infection [11], [23], [26]. The common assays applied to quantify SHR rely on the precise restoration of transgenic marker genes from two incomplete but overlapping parts [9], [14]. Thereby, SHR measurements can be performed without interfering with essential functions of endogenous genes and can take place under well-defined conditions. Indeed, multiple previous publications demonstrate a reproducible increase of SHR reporter lines in response to different stimuli (Lebel et al., 1993; Ries et al., 2000b; Lucht et al., 2002; Kovalchuk et al., 2003; Molinier et al., 2005; Boyko et al., 2006b). However, upon direct comparison of recombination frequencies between two different SHR trap lines under a broad variety of stress treatments, it becomes very obvious that the lines exhibit different sensitivities and features in stress response. Upon the systematic application of ten different abiotic stress types, line 11 is very responsive to all stresses except for high doses of paraquat, all doses of UV-C and histone deacetylase inhibitors. Line 1445 generally shows a higher level of stimulation and does not respond to the doses of salt, cold and paraquat applied here. Such differences can be due to features of the SHR trap construction (type of recombination, length of the homology overlap), the genomic position or chromatin configuration of the transgene, the ecotype background or a combination of several or all of these parameters. Some are conceivable, e.g. the weaker reactivity of line 1445 to salt and cold since its background ecotype Col is less sensitive to both stresses than C24 of line 11 [42], [43]. Similarly, the baseline of SHR in the non-stressed lines correlates with the length of the overlap, as described previously for extrachromosomal [44], [45] or intrachromosomal [46] recombination. Although both lines in our study carry SHR traps at similar gene-rich euchromatic genomic locations (bottom arm of chromosome 2; 2.7 Mbp apart), line 11 with a homologous overlap of 1213 bp has, on average, five times more SHR events than line 1445 with only a 618 bp overlap. Lines with a low SHR baseline (≤0.2 events per plant; lines 651, IC9, 1445) generally have a greater dynamic range in response compared to line 11. Notably, and irrespective of the background frequency, all lines seem to reach similar absolute numbers of SHR events (a maximum of 30–40 events in individual plants). This suggests an upper limit for SHR in Arabidopsis. Since the plants with maximum spot numbers display strong developmental retardation, it is likely that higher doses of DNA damage would lead to accumulation of mutations and apoptosis. However, it should be emphasized that a lack of transgenerational effect is neither due to ineffective nor to injurious S0 treatments. The different SHR-inducing doses applied, e.g. for paraquat and bleocin, cover a range from having no phenotypic effect to slight or severe growth retardation.

Other differences in the response of the lines are less plausible. Line 11, responding to UV-C in many independent studies [18], [23] and stimulated in our hands by many other stress types, did not show a significant increase in SHR, although the efficacy of the treatment was obvious from the response in the other lines. These differences indicate a role of additional factors controlling SHR and determined by conditions other than only the stress treatment.

### Efficiency and mechanisms of stress responses

Each treatment caused a significant increase in at least one of the two extensively tested SHR trap lines. The results confirm previous data [23], [26] about increased SHR after salt stress in line 11 and a lack of response in line 651. There was also no SHR stimulation by salt in Columbia-based lines IC9 and 1445, probably due to the higher resistance of Col to salt stress [42].

The general efficacy of heat may be mediated by affecting chromatin condensation and accessibility of DNA for recombination because it leads not only to SHR induction in all tested lines [9], [11; this study] but also to reactivation or enhanced transcription from epigenetically silenced repetitive elements and transgenes in Arabidopsis [47; GENAU consortium, unpublished data].

Paraquat and bleocin are both known to produce reactive oxygen species (ROS). While bleocin leads to highly increased SHR in both tested lines, we found that lower concentrations of paraquat stimulated SHR only in line 11, and higher concentrations suppressed SHR in both lines in three independent experiments. Higher SHR stimulation by bleocin is likely due to its pronounced single and double strand break induction [48] compared to paraquat-induced accumulation of ROS species mainly in chloroplasts [49].

The lack of response in line 11 after UV-C irradiation, so far repeatedly described to be effective [18], [23], is certainly not due to an insufficient dose since the same conditions of irradiation caused a significant SHR increase in lines 651, IC9 and 1445 (Table 1 and Table S17). Transcript levels of marker genes that are indicative for induced homologous recombination were found to be elevated after UV-C treatment to a similar level in all lines, including line 11 (Figure 1 and Figure S1). We can only assume unknown differences in the UV sources used and/or conditions during or after application that elude experimental control to be responsible for the discrepancy with the published data. Thus, the results suggest that not all recombinogenic regions respond equally to the induction of the homologous recombination pathway.

Zebularine has never been tested on SHR before, and its mode of action in recombination induction is not known. We first suspected the increased SHR to be due to a DNA demethylation effect [27] on the SHR trap inserts, but this is unlikely since the transgene promoters are not hypermethylated (MR, AP, OMS, unpublished data). On the other hand, zebularine may demethylate and activate SHR genes whose gene products could be limiting factors of recombination. The most likely scenario is that the incorporation of the cytosine analog into DNA and the covalent binding of DNA methyltransferases, by which it exerts its demethylating activity [50], blocks the replication machinery or may cause DNA breaks that have to be repaired by homologous recombination.

The other chromatin-affecting drugs, trichostatin A and sodium butyrate, lead to generally enhanced transcription via inhibition of histone deacetylases (HDACs) and subsequent histone hyperacetylation. Therefore, it is possible that both drugs directly or indirectly stimulate transcription of factors in the SHR pathway. Alternatively, they could render the chromatin around the SHR traps more accessible for recombination or change the chromatin-associated proteins that interact with the modified histone tails.

In synopsis, for the applied treatments, we obtained responses ranging from a significant decrease of SHR after high doses of paraquat to up to a 150-fold increase after zebularine administration. The strongest response, over 100-fold stimulation, could only be achieved with drugs directly affecting DNA (bleocin and zebularine). A similar increase of SHR was observed in the mutants *FAS1* and *FAS2* that have defective subunits of the Chromatin Assembly Factor-1 (CAF-1) [20], [21]. This multiprotein factor is responsible for nucleosome assembly onto DNA during replication [51]. There could be several reasons for an increased SHR in *fas* mutants, such as global chromatin decondensation [21], a generally enhanced expression of SHR genes in these mutant backgrounds [20] and/or crosstalk between DNA replication and DNA repair mechanisms [52]. The significant effects of chromatin-modifying treatments are plausible considering that homologous recombination inevitably includes a screen for sequence similarity. It is hard to imagine that this can be efficiently achieved without modifying the higher order chromatin structure of participating DNA molecules. Indeed, data from many systems suggest marking of broken DNA with specific chromatin modifications, recruitment of repair proteins by these marks and assistance by chromatin remodeling factors in repair [for reviews see 53], [54]. Beside the proteins already mentioned, several plant chromatin remodeling factors determine the rate of homologous recombination [55], [56] and indicate a considerable role of DNA accessibility in the control of homologous recombination.

### Rare and stochastic transgenerational effects

The multiple conditions that induce a significant increase of SHR in the treated generations, assumed to imply different perception and signaling pathways, provided a solid basis to study transmission of the stress effects into subsequent generations. With very few exceptions, progeny populations did not show a significantly increased SHR in S1, although the SHR induction by bleocin and zebularine in S0 had been much higher than described previously for other treatments. The rare exceptions, partially with a modest level of statistical significance, were treatments with a low dose of paraquat and an intermediate dose of zebularine for line 11, as well as a low dose of bleocin for line 1445. However, repetition of the paraquat experiment, which had a good level of confidence for the increase in the first data set, did not confirm the response under apparently identical conditions. The other two observed cases of transgenerational effects rather seemed to indicate experimental variation in the baseline of SHR, since lower and higher doses of the same treatments had no effect. The ambiguity of S1 effects is further confirmed by the data from S2, which completely lacked significantly increased SHR values that were expected on the basis of the dominant trait that was stable over at least four generations in previous experiments [18]. The data obtained by the analysis of individual recombination events using SHR trap lines are in line with the expression of several genes known to encode for protein factors involved in homologous recombination. Therefore, under our experimental conditions, transgenerational stress effects on SHR seem to occur in a rather stochastic manner, independent of the initial degree of stimulation, and are not a general strategy to respond to abiotic stress in Arabidopsis. It is possible that the defined physical or chemical treatments are just one part of a gating function together with other extraneous triggers or internal latches. As long as these are not intelligible, experimental approaches to understand the molecular mechanism of transgenerational stress effects remain difficult. However, stochastic responses are part of evolutionary successful adaptation mechanisms in irregularly changing environments (for review see [57]) and it is conceivable that plants have adopted similar strategies.

## Materials and Methods

### Plant material

SHR trap line 11 [14], [58] carries direct and line 1445 [22], [59] inverted, incomplete and overlapping repeats of the transgenic reporter construct, from which a functional β-glucuronidase (GUS) gene can be restored by intramolecular homologous recombination. The position of the T-DNA insertion was mapped in both lines to the bottom (long) arm of chromosome 2. The exact nucleotide positions are 11765197 for line 11 [60] and 14424870 for line 1445. Seeds for all S0 experiments originated from the same batch of seeds amplified from a single plant. All seed batches were genotyped for homozygosity of the specific recombination traps.

### Stress assays

All stress assays were carried out under long day conditions (16 h light/8 h dark) at 22°C and *in vitro* (with the exception of soil-grown UV-B stressed plants, see below). For salt, osmotic and oxidative stress, plants were initially grown on ½ MS. Twelve day old plantlets were transferred to media containing 100 mM sodium chloride (Sigma-Aldrich), 100 mM mannitol (Sigma-Aldrich), and paraquat (dimethyl viologen, Sigma) at concentrations between 0.1 and 1 µM, grown for another 5 days and stained for GUS activity immediately (without recovery). For freezing and heat stress, plants were initially grown as described above. On day 12, the plates with plants were transferred to either −4°C or 37°C chambers for 24 h. After the temperature stress, plants were grown for another 4 days under non-stress conditions for recovery and stained for GUS activity on day 17. For bleocin (commercial name for bleomycin; Calbiochem) treatment, seeds were germinated on drug-containing GM media, grown without any transfer and stained on day 17. To analyze the responses to HDAC inhibitors, plants were germinated in the presence of either 1 µg/ml of trichostatin A (Sigma) or 0.4 mM sodium butyrate (Fluka). After 10 days, seedlings were stained for GUS activity. For UV-B experiments, *in vitro* grown seedlings (10 to 12 days old) were transferred onto soil and grown for another 4 days prior to transfer into a UV-B growth chamber (Percival, USA) equipped with Philips TL 20W/12RS lamps. After daily exposure to UV-B dosages equivalent to 3.1, 4.7 and 6.3 kJ/m^2^ for a total of 24 days, plants were allowed to set seeds without irradiation. GUS activity was then scored in 14 day old progeny seedlings; siblings were grown on soil to generate S2 seeds. For analysis of the SHR frequency in S0, plants were grown and treated as described above, but GUS staining was performed on UV-B-exposed seedlings after 8 days. UV-C irradiation (254 nm) was applied in doses of either 750, 1500 or 3000 J/m^2^ to 11 day old plants grown on GM plates using a UV crosslinker (Stratalinker 2400). For the zebularine treatments, seeds were germinated in aqueous solutions of 20, 40 or 80 µM zebularine for three days. The seedlings were transferred to drug-free solid GM media and stained for GUS activity after two weeks of recovery.

From each stressed population as well as from non-treated siblings (mock S0), five randomly selected plants were transferred to soil and grown to obtain seeds. Unless stated otherwise, seeds were harvested in pools, surface-sterilized, grown *in vitro* for 17 days and stained for GUS activity to analyze SHR frequencies in the non-stressed S1 and S2 generations. For a more detailed description of stress treatments, see Text S1.

### GUS assay

GUS staining solution (1 mM sodium phosphate buffer, pH 7; 10 mM EDTA, 0.1% Triton-X, 100 µg/ml chloramphenicol; 2 mM potassium ferrocyanide; 2 mM potassium ferricyanide; 0.5 mg/ml X-glucuronide) was infiltrated into submerged plants by vacuum (15 min). Plants were incubated overnight at 37°C and de-stained by several overnight washings with 70% ethanol. SHR events, indicated by blue cells or sectors, were evaluated under a stereomicroscope (Leica).

### Reverse transcription and real time PCR

RNA was prepared using RNeasy Plant Mini Kit (Qiagen) according to the manufacturer's instructions. To remove residual DNA contamination, 1 µg of total RNA was treated with 50 units of DNase I (Fermentas) for 30 min. The absence of contamination with genomic DNA was confirmed using samples lacking the reverse transcriptase (RT) by conventional PCR with primers specific for the gene *UBC28* (At1g64230), also used for normalization: UBC28qF (5′-TCC AGA AGG ATC CTC CAA CTT CCT GCA GT-3′) and UBC28qR (5′-ATG GTT ACG AGA AAG ACA CCG CCT GAA TA-3′) (40 cycles). cDNA was produced using Revert Aid H Minus M-MuLV RT and Random hexamer primers (Fermentas) according to the manufacturer's instructions.

Real-time PCR was run on an iQ5 light cycler (Bio-Rad) using 2× SensiMix Plus SYBR Kit & Fluorescein kit (Peqlab). Genes were analyzed for their expression using the following primers: *RAD51* (At5g20850): AtRAD51fwd (5′-CTC CGA GGA AGG ATC TCT TGC AG-3′) and AtRAD51rev (5′-GCT CGC ACT AGT GAA CCC CAG AGG-3′); *BRCA1* (At4g21070): BRCA1qF (5′-GTT ACG TGT GCA AAA CTC ATA CCA GAA TG-3′) and BRCA1qR (5′-GAT ACT TGT TTA GGC TGA GAG TGC AGT GG-3′) and *MIM* (At5g61460): MIMqF (5′-TTT CGT GGG CCA GTT CAG ACT ACT CTT-3′), MIMqR (5′-CTC AAG ATT CTC CTC TGC CTC CCT CTT-3′); *ATM* (At3g48190): ATMqF (5′-CAT CTT CGA CGA AAT CTT CTT AGA GCA GT-3′) and ATMqR (5′-ACA GAC ATC CCA TTG AGA TGG TGT TGG A-3′). All were normalized to the reference gene *UBC28* whose expression did not change significantly under any of the conditions applied here. Fluorescence data were acquired at 73°C for *UBC28*, at 75°C for *RAD51*, *BRCA1* and *ATM*, and at 76°C for *MIM*, to avoid signals from possible primer dimers.

Data analysis and assembly were performed with Bio-Rad iQ5 software (Bio-Rad), Excel (Microsoft) and SigmaPlot (SPSS Science Software).

## Supporting Information

Text S1 (0.03 MB DOC)

Figure S1 Expression of SHR genes after UV-C irradiation
(0.19 MB TIF)

Table S1 The effect of salt stress on the frequency of SHR
(0.06 MB DOC)

Table S2 The effect of osmotic (mannitol) stress on the frequency of SHR
(0.06 MB DOC)

Table S3 The effect of cold stress on the frequency of SHR
(0.06 MB DOC)

Table S4 The effect of heat stress on the frequency of SHR
(0.09 MB DOC)

Table S5 The effect of radiomimetic (bleocin) stress on the frequency of SHR in the S0 generation
(0.06 MB DOC)

Table S6 The effect of radiomimetic (bleocin) stress on the frequency of SHR in the S1 generation
(0.06 MB DOC)

Table S7 The effect of radiomimetic (bleocin) stress on the frequency of SHR in the S2 generation
(0.06 MB DOC)

Table S8 The effect of oxidative (paraquat) stress on the frequency of SHR
(0.09 MB DOC)

Table S9 The effect of paraquat stress on the frequency of SHR in the progeny of individual S0 plants
(0.05 MB DOC)

Table S10 The effect of UV-B stress on the frequency of SHR
(0.06 MB DOC)

Table S11 The effect of UV-C stress on the frequency of SHR
(0.08 MB DOC)

Table S12 The effect of DNA demethylation (zebularine) stress on the frequency of SHR in the S0 generation
(0.08 MB DOC)

Table S13 The effect of DNA demethylation (zebularine) stress on the frequency of SHR in the S1 generation
(0.10 MB DOC)

Table S14 The effect of DNA demethylation (zebularine) stress on the frequency of SHR in the S2 generation
(0.08 MB DOC)

Table S15 The effect of histone hyperacetylation by trichostatin A (TSA) stress on the frequency of SHR
(0.06 MB DOC)

Table S16 The effect of histone hyperacetylation (sodium butyrate) stress on the frequency of SHR
(0.06 MB DOC)

Table S17 The effect of UV-C stress on the frequency of SHR in four different SHR trap lines
(0.08 MB DOC)
