# Supplementary material for: Transgenerational Stress Memory Is Not a General Response in Arabidopsis
Source: PLoS One. 2009 Apr 21;4(4):e5202. doi: 10.1371/journal.pone.0005202 (PMC2668180; doi:10.1371/journal.pone.0005202)
Supplement: Text S1 — (0.03 MB DOC) [file pone.0005202.s001.doc]

**Text S1**

**Supporting information**

**Supplementary plant material**

Line 651 [14, 23] harbors an inverted recombination substrate and requires intramolecular recombination. The T-DNA insert was mapped to the pericentromeric region of chromosome 5 at nucleotide position 10455625 [60]. Line IC9 [25] contains direct repeats that require intermolecular recombination to restore a functional marker gene and carries the transgene on the top arm of chromosome 5 at nucleotide position 8633790 (Frank Hartung and Holger Puchta, personal communication). Line 651 is in the C24 background while line IC9 is in Columbia. Both lines carry single transgene copies.

**Supplementary results**

**Experiments with additional SHR tester lines**

In addition to the systematic experiments with lines 11 and 1445, we also tested individual treatments (heat and zebularine) on lines 651 and IC9 and on their S1 and S2 progeny. Heat-stressed S0 populations of 651 and IC9 showed significant 2.6- and 5.9-fold increases in SHR (*P<0.05*), respectively (Table S4). As for lines 11 and 1445, SHR in S1 and S2 post-stress populations did not significantly differ from mock values. Zebularine increased SHR approximately 40-fold in lines 651 and 40- to 80-fold in line IC9 (Table S12). Initial assays of SHR frequencies in S1 populations from line 651 indicated a highly significant 10-15-fold increased SHR compared to mock-treated S1 (Table S13). This could have suggested a strong induction of transgenerational stress memory in this line after zebularine application. However, another S0 mock population (grown in parallel with the S1 populations) had an 8.7-fold higher baseline than the S1 mock and a similar SHR frequency as the zebularine-stressed samples (1.7-, 1.1- and 1.3-fold increase after 20, 40 and 80 µM zebularine treatment, respectively, Table S13). Since both mocks originated from the same seed batch, were grown in parallel and differed only in one more generation, the difference in recombination response between them was difficult to explain. To test the reproducibility of the SHR induction in line 651, we generated another batch of S1 progeny from line 651 plants stressed in S0 by zebularine and tested their SHR frequency. This repetition did not reveal any significant SHR increase between mock and zebularine-stressed plants, indicating that the initially observed difference was indeed due to (a) factor(s) different from transgenerational memory. Zebularine-treated plants of line IC9 never showed any significant difference compared to mock values in the two experimental repetitions performed.

**UV-C irradiation activates genes involved in SHR but does not lead to increased recombination at the reporter locus in line 11**

Several recombination trap lines were previously reported to show an increased number of SHR events upon UV-C treatment, including line 11 [18, 23]. However, in our experiments, UV-C stressed line 1445, but not, line 11 showed significantly elevated numbers of GUS spots in S0 (Table 1, and additional three biological replicates not shown). This suggests that different lines might have specific responsiveness to UV-C. To test this, we scored the SHR frequency of all four SHR trap lines 651, 11, IC9 and 1445 after treatment with several doses of UV-C in parallel to measuring induction levels of SHR marker genes. Quantification of GUS spots revealed significantly increased SHR in 651, IC9 and 1445, but not in 11 (Table S17), confirming results of our previous experiments and indeed suggesting line-specific responsiveness. *RAD51* and *MIM* genes, indicative of ongoing DNA repair by SHR, were induced to a similar degree in all lines, including line 11, after all UV-C doses applied (Figure S1). Therefore, the lack of recombination increase for this specific line (reacting well to many other treatments, Table 1) can not be due to non-operational stress treatment in our experiments. While UV-C irradiation effectively induced DNA repair mechanisms, it is not inevitably connected with stimulation of SHR at all recombinogenic regions.

**Legend for Supplementary Figure S1: Expression of SHR genes after UV-C irradiation.** Relative expression of *RAD51* (A) and *MIM* (B) was analyzed 48h after UV-C stress by real-time PCR (with *UBC28* as a reference gene that is not influenced by any treatment). The amount of transcript in stressed samples was normalized to that in mock-treated sample for each line. Both genes were significantly induced by all UV-C doses in all tested lines.
