# Supplementary figures and images for: Transgenerational Stress Memory Is Not a General Response in Arabidopsis
Source: PLoS One. 2009 Apr 21;4(4):e5202. doi: 10.1371/journal.pone.0005202 (PMC2668180; doi:10.1371/journal.pone.0005202)

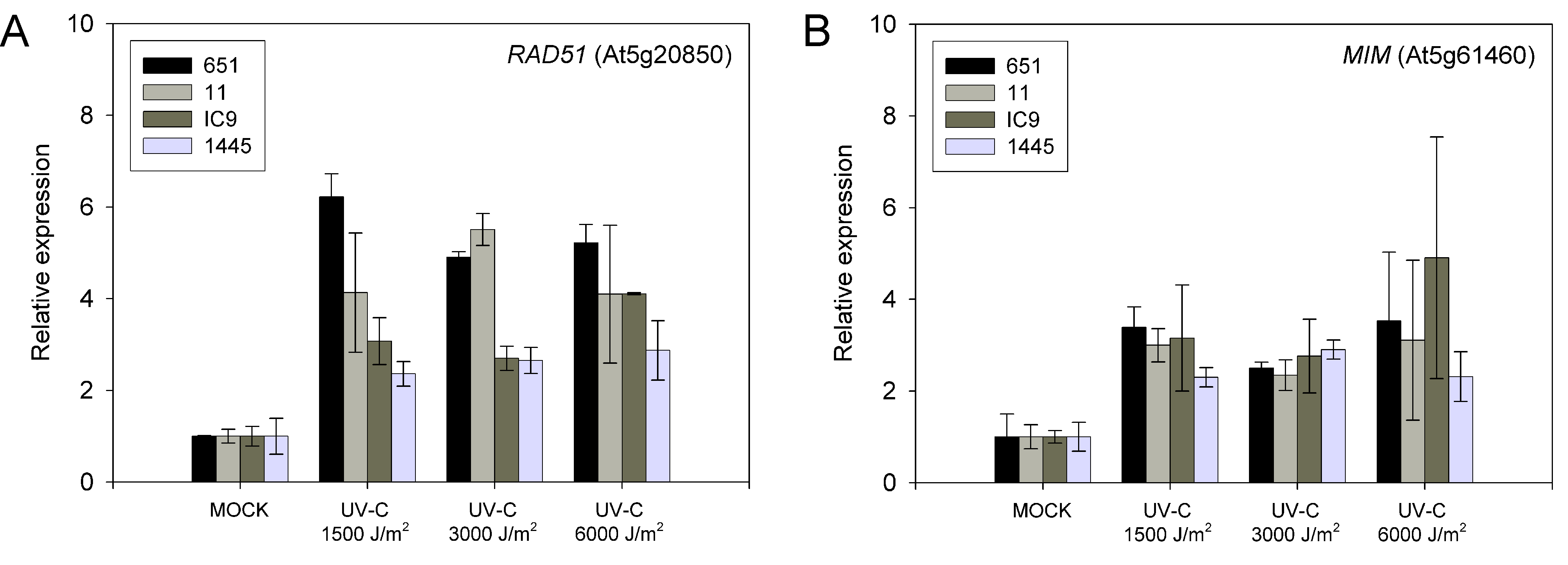

Supplement: Figure S1 — Expression of SHR genes after UV-C irradiation (0.19 MB TIF) [file pone.0005202.s002.tif]
