# Supplementary material for: Transgenerational Stress Memory Is Not a General Response in Arabidopsis
Source: PLoS One. 2009 Apr 21;4(4):e5202. doi: 10.1371/journal.pone.0005202 (PMC2668180; doi:10.1371/journal.pone.0005202)
Supplement: Table S1 — The effect of salt stress on the frequency of SHR (0.06 MB DOC) [file pone.0005202.s003.doc]

**Supplementary Table 1: The effect of salt stress on the frequency of SHR**

| Generation |  | S0 | S0 | S1 | S1 | S2 | S2 |
| --- | --- | --- | --- | --- | --- | --- | --- |
| Pre-growth | Medium | 1/2 MS | 1/2 MS | 1/2 MS | 1/2 MS | GM | GM |
|  | Day length | 16 h | 16 h | 16 h | 16 h | 16 h | 16 h |
|  | Temperature | 22°C | 22°C | 22°C | 22°C | 22°C | 22°C |
|  | Duration | 12 d | 12 d | 17 d | 17 d | 17 d | 17 d |
|  | Transplanted | yes | yes | no | no | no | no |
| Stress | Treatment | **MOCK S0** | **100 mM NaCl S0** | **MOCK S1** | **100 mM NaCl S1** | **MOCK S2** | **100 mM NaCl S2** |
|  | Duration of treatment | none | 5 d | none | none | none | none |
|  | Recovery | none | none | none | none | none | none |
| **11** | Analyzed plants | 168 | 164 | 415 | 398 | 396 | 472 |
|  | Recombination (GUS spots) | 163 | 479 | 359 | 349 | 1298 | 1178 |
|  | GUS spots/plant | 0.970 | 2.921 | 0.865 | 0.877 | 3.278 | 2.496 |
|  | Normalized recombination | 1.000 | 3.010 | 1.000 | 1.014 | 1.000 | 0.761 |
|  | Fold change |  | 3.0 |  | 1.0 |  | 0.8 |
|  | Fisher's exact test (P value) |  | 0.0001 |  | 0.9181 |  | 0.0006 |
| **1445** | Analyzed plants | 44 | 45 |  |  |  |  |
|  | Recombination (GUS spots) | 5 | 8 |  |  |  |  |
|  | GUS spots/plant | 0.114 | 0.178 |  |  |  |  |
|  | Normalized recombination | 1.000 | 1.564 |  |  |  |  |
|  | Fold change |  | 1.6 |  |  |  |  |
|  | Fisher's exact test (P value) |  | 0.5590 |  |  |  |  |
