# Supplementary material for: Transgenerational Stress Memory Is Not a General Response in Arabidopsis
Source: PLoS One. 2009 Apr 21;4(4):e5202. doi: 10.1371/journal.pone.0005202 (PMC2668180; doi:10.1371/journal.pone.0005202)
Supplement: Table S2 — The effect of osmotic (mannitol) stress on the frequency of SHR (0.06 MB DOC) [file pone.0005202.s004.doc]

**Supplementary Table 2: The effect of osmotic (mannitol) stress on the frequency of SHR**

| Generation |  | S0 | S0 | S1 | S1 | S2 | S2 |
| --- | --- | --- | --- | --- | --- | --- | --- |
| Pre-growth | Medium | 1/2 MS | 1/2 MS | 1/2 MS | 1/2 MS | 1/2 MS | 1/2 MS |
|  | Day length | 16 h | 16 h | 16 h | 16 h | 16 h | 16 h |
|  | Temperature | 22°C | 22°C | 22°C | 22°C | 22°C | 22°C |
|  | Duration | 12 d | 12 d | 21 d | 21 d | 21 d | 21 d |
|  | Transplanted | yes | yes | no | no | no | no |
| Stress | Treatment | **MOCK S0** | **100 mM mannitol S0** | **MOCK S1** | **100 mM mannitol S1** | **MOCK S2** | **100 mM mannitol S2** |
|  | Duration of treatment | none | 5 d | none | none | none | none |
|  | Recovery | none | none | none | none | none | none |
| **11** | Analyzed plants | 124 | 129 | 327 | 368 | 345 | 473 |
|  | Recombination (GUS spots) | 88 | 190 | 293 | 185 | 1201 | 890 |
|  | GUS spots/plant | 0.710 | 1.473 | 0.896 | 0.503 | 3.481 | 1.882 |
|  | Normalized recombination | 1.000 | 2.075 | 1.000 | 0.561 | 1.000 | 0.541 |
|  | Fold change |  | 2.1 |  | 0.6 |  | 0.5 |
|  | Fisher's exact test (P value) |  | 0.0001 |  | 0.0001 |  | 0.0001 |
| **1445** | Analyzed plants | 89 | 78 |  |  |  |  |
|  | Recombination (GUS spots) | 152 | 127 |  |  |  |  |
|  | GUS spots/plant | 1.707 | 1.628 |  |  |  |  |
|  | Normalized recombination | 1.0000 | 0.9537 |  |  |  |  |
|  | Fold change |  | 1.0 |  |  |  |  |
|  | Fisher's exact test (P value) |  | 1.0000 |  |  |  |  |
