# Supplementary material for: Transgenerational Stress Memory Is Not a General Response in Arabidopsis
Source: PLoS One. 2009 Apr 21;4(4):e5202. doi: 10.1371/journal.pone.0005202 (PMC2668180; doi:10.1371/journal.pone.0005202)
Supplement: Table S3 — The effect of cold stress on the frequency of SHR (0.06 MB DOC) [file pone.0005202.s005.doc]

**Supplementary Table 3: The effect of cold stress on the frequency of SHR**

| Generation |  | S0 | S0 | S1 | S1 | S2 | S2 |
| --- | --- | --- | --- | --- | --- | --- | --- |
| Pre-growth | Medium | 1/2 MS | 1/2 MS | 1/2 MS | 1/2 MS | GM | GM |
|  | Day length | 16 h | 16 h | 16 h | 16 h | 16 h | 16 h |
|  | Temperature | 22°C | 22°C | 22°C | 22°C | 22°C | 22°C |
|  | Duration | 17 d | 17 d | 17 d | 17 d | 17 d | 17 d |
|  | Transplanted | yes | yes | no | no | no | no |
| Stress | Treatment | **MOCK S0** | **-4°C S0** | **MOCK S1** | **-4°C S1** | **MOCK S2** | **-4°C S2** |
|  | Duration of treatment | none | 24 h | none | none | none | none |
|  | Recovery | none | none | none | none | none | none |
| **11** | Analyzed plants | 44 | 51 | 88 | 81 | 51 | 54 |
|  | Recombination (GUS spots) | 75 | 195 | 66 | 79 | 97 | 94 |
|  | GUS spots/plant | 1.705 | 3.824 | 0.750 | 0.975 | 1.902 | 1.741 |
|  | Normalized recombination | 1.000 | 2.243 | 1.000 | 1.300 | 1.000 | 0.915 |
|  | Fold change |  | 2.2 |  | 1.3 |  | 0.9 |
|  | Fisher's exact test (P value) |  | 0.0014 |  | 0.2590 |  | 0.8080 |
| **1445** | Analyzed plants | 44 | 48 |  |  |  |  |
|  | Recombination (GUS spots) | 5 | 4 |  |  |  |  |
|  | GUS spots/plant | 0.114 | 0.083 |  |  |  |  |
|  | Normalized recombination | 1.000 | 0.733 |  |  |  |  |
|  | Fold change |  | 0.7 |  |  |  |  |
|  | Fisher's exact test (P value) |  | 0.7360 |  |  |  |  |
