# Supplementary material for: Transgenerational Stress Memory Is Not a General Response in Arabidopsis
Source: PLoS One. 2009 Apr 21;4(4):e5202. doi: 10.1371/journal.pone.0005202 (PMC2668180; doi:10.1371/journal.pone.0005202)
Supplement: Table S4 — The effect of heat stress on the frequency of SHR (0.09 MB DOC) [file pone.0005202.s006.doc]

**Supplementary Table 4: The effect of heat stress on the frequency of SHR**

| Generation |  | S0 | S0 | S1 | S1 | S2 | S2 |
| --- | --- | --- | --- | --- | --- | --- | --- |
| Pre-growth | Medium | 1/2 MS | 1/2 MS | 1/2 MS | 1/2 MS | GM | GM |
|  | Day length | 16 h | 16.h | 16 h | 16 h | 16 h | 16 h |
|  | Temperature | 22°C | 22°C | 22°C | 22°C | 22°C | 22°C |
|  | Duration | 12 d | 12.d | 17 d | 17 d | 17 d | 17 d |
|  | Transplanted | yes | yes | no | no | no | no |
| Stress | Treatment | **MOCK S0** | **37°C S0** | **MOCK S1** | **37°C S1** | **MOCK S2** | **37°C S2** |
|  | Duration of treatment | none | 24.h | none | none | none | none |
|  | Recovery | none | 5.d | none | none | none | none |
| **651** | Analyzed plants | 45 | 44 | 73 | 73 | 54 | 51 |
|  | Recombination (GUS spots) | 7 | 18 | 1 | 1 | 6 | 4 |
|  | GUS spots/plant | 0.156 | 0.409 | 0.014 | 0.014 | 0.111 | 0.078 |
|  | Normalized recombination | 1.000 | 2.630 | 1.000 | 1.000 | 1.000 | 0.706 |
|  | Fold change |  | 2.6 |  | 1.0 |  | 0.7 |
|  | Fisher's exact test (P value) |  | 0.0680 |  | 1.0000 |  | 0.7450 |
| **11** | Analyzed plants | 44 | 46 | 98 | 81 | 52 | 51 |
|  | Recombination (GUS spots) | 75 | 221 | 48 | 21 | 55 | 66 |
|  | GUS spots/plant | 1.705 | 4.804 | 0.490 | 0.259 | 1.058 | 1.294 |
|  | Normalized recombination | 1.000 | 2.819 | 1.000 | 0.529 | 1.000 | 1.224 |
|  | Fold change |  | 2.8 |  | 0.5 |  | 1.2 |
|  | Fisher's exact test (P value) |  | 0.0001 |  | 0.0434 |  | 0.5030 |
| **IC9** | Analyzed plants | 80 | 95 | 55 | 60 | 59 | 61 |
|  | Recombination (GUS spots) | 2 | 14 | 11 | 23 | 4 | 3 |
|  | GUS spots/plant | 0.025 | 0.147 | 0.200 | 0.383 | 0.068 | 0.049 |
|  | Normalized recombination | 1.000 | 5.895 | 1.000 | 1.917 | 1.000 | 0.725 |
|  | Fold change |  | 5.9 |  | 1.9 |  | 0.7 |
|  | Fisher's exact test (P value) |  | 0.0151 |  | 0.1210 |  | 0.7180 |
| **1445** | Analyzed plants | 44 | 47 | 80 | 81 | 61 | 63 |
|  | Recombination (GUS spots) | 5 | 18 | 5 | 5 | 15 | 14 |
|  | GUS spots/plant | 0.114 | 0.383 | 0.063 | 0.062 | 0.246 | 0.222 |
|  | Normalized recombination | 1.000 | 3.370 | 1.000 | 0.988 | 1.000 | 0.904 |
|  | Fold change |  | 3.4 |  | 1.0 |  | 0.9 |
|  | Fisher's exact test (P value) |  | 0.0325 |  | 1.0000 |  | 0.8390 |
