# Supplementary material for: Transgenerational Stress Memory Is Not a General Response in Arabidopsis
Source: PLoS One. 2009 Apr 21;4(4):e5202. doi: 10.1371/journal.pone.0005202 (PMC2668180; doi:10.1371/journal.pone.0005202)
Supplement: Table S5 — The effect of radiomimetic (bleocin) stress on the frequency of SHR in the S0 generation (0.06 MB DOC) [file pone.0005202.s007.doc]

**Supplementary Table 5: The effect of radiomimetic (bleocin) stress on the frequency of SHR in the S0 generation**

| Generation |  | S0 | S0 | S0 | S0 | S0 | S0 | S0 |
| --- | --- | --- | --- | --- | --- | --- | --- | --- |
| Pre-growth | Medium | GM | GM | GM | GM | GM | GM | GM |
|  | Day length | 16 h | 16 h | 16 h | 16 h | 16 h | 16 h | 16 h |
|  | Temperature | 22°C | 22°C | 22°C | 22°C | 22°C | 22°C | 22°C |
|  | Duration | none | see stress | see stress | see stress | see stress | see stress | see stress |
|  | Transplanted | no | no | no | no | no | no | no |
| Stress | Treatment | **MOCK S0** | **10 ng/ml bleocin S0** | **20 ng/ml bleocin S0** | **50 ng/ml bleocin S0** | **100 ng/ml bleocin S0** | **200 ng/ml bleocin S0** | **400 ng/ml bleocin S0** |
|  | Duration of treatment | none | 17 d | 17 d | 17 d | 17 d | 17 d | 17 d |
|  | Recovery | none | none | none | none | none | none | none |
| **11** | Analyzed plants | 51 | 50 | 47 | 52 | 49 | 51 | 51 |
|  | Recombination (GUS spots) | 87 | 246 | 166 | 263 | 228 | 370 | 319 |
|  | GUS spots/plant | 1.706 | 4.920 | 3.532 | 5.058 | 4.653 | 7.255 | 6.255 |
|  | Normalized recombination | 1.000 | 2.884 | 2.070 | 2.965 | 2.728 | 4.253 | 3.667 |
|  | Fold change |  | 2.9 | 2.1 | 3.0 | 2.7 | 4.3 | 3.7 |
|  | Fisher's exact test (P value) |  | 0.0001 | 0.0033 | 0.0001 | 0.0001 | 0.0001 | 0.0001 |
| **1445** | Analyzed plants | 55 | 50 | 50 | 55 | 51 | 50 | 51 |
|  | Recombination (GUS spots) | 7 | 98 | 133 | 158 | 340 | 349 | 738 |
|  | GUS spots/plant | 0.127 | 1.960 | 2.660 | 2.873 | 6.667 | 6.980 | 14.471 |
|  | Normalized recombination | 1.000 | 15.400 | 20.900 | 22.571 | 52.381 | 54.843 | 113.697 |
|  | Fold change |  | 15.4 | 20.9 | 22.6 | 52.4 | 54.8 | 113.7 |
|  | Fisher's exact test (P value) |  | 0.0001 | 0.0001 | 0.0001 | 0.0001 | 0.0001 | 0.0001 |
