# Supplementary material for: Transgenerational Stress Memory Is Not a General Response in Arabidopsis
Source: PLoS One. 2009 Apr 21;4(4):e5202. doi: 10.1371/journal.pone.0005202 (PMC2668180; doi:10.1371/journal.pone.0005202)
Supplement: Table S6 — The effect of radiomimetic (bleocin) stress on the frequency of SHR in the S1 generation (0.06 MB DOC) [file pone.0005202.s008.doc]

**Supplementary Table 6: The effect of radiomimetic (bleocin) stress on the frequency of SHR in the S1 generation**

| Generation |  | S1 | S1 | S1 | S1 | S1 | S1 | S1 |
| --- | --- | --- | --- | --- | --- | --- | --- | --- |
| Pre-growth | Medium | GM | GM | GM | GM | GM | GM | GM |
|  | Day length | 16 h | 16 h | 16 h | 16 h | 16 h | 16 h | 16 h |
|  | Temperature | 22°C | 22°C | 22°C | 22°C | 22°C | 22°C | 22°C |
|  | Duration | 17 d | 17 d | 17 d | 17 d | 17 d | 17 d | 17 d |
|  | Transplanted | no | no | no | no | no | no | no |
| Stress | Treatment | **MOCK bleocin S1** | **10 ng/ml bleocin S1** | **20 ng/ml bleocin S1** | **50 ng/ml bleocin S1** | **100 ng/ml bleocin S1** | **200 ng/ml bleocin S1** | **400 ng/ml bleocin S1** |
|  | Duration of treatment | none | none | none | none | none | none | none |
|  | Recovery | none | none | none | none | none | none | none |
| **11** | Analyzed plants | 52 | 52 | 52 | 55 | 53 | 54 | 51 |
|  | Recombination (GUS spots) | 54 | 76 | 61 | 69 | 61 | 60 | 92 |
|  | GUS spots/plant | 1.038 | 1.462 | 1.173 | 1.255 | 1.151 | 1.111 | 1.804 |
|  | Normalized recombination | 1.000 | 1.407 | 1.130 | 1.208 | 1.108 | 1.070 | 1.737 |
|  | Fold change |  | 1.4 | 1.1 | 1.2 | 1.1 | 1.1 | 1.7 |
|  | Fisher's exact test (P value) |  | 0.2344 | 0.6860 | 0.5089 | 0.7872 | 0.8927 | 0.0379 |
| **1445** | Analyzed plants | 56 | 59 | 53 | 51 | 51 | 54 | 54 |
|  | Recombination (GUS spots) | 16 | 29 | 21 | 33 | 28 | 27 | 15 |
|  | GUS spots/plant | 0.286 | 0.492 | 0.396 | 0.647 | 0.549 | 0.500 | 0.278 |
|  | Normalized recombination | 1.000 | 1.720 | 1.387 | 2.265 | 1.922 | 1.750 | 0.972 |
|  | Fold change |  | 1.7 | 1.4 | 2.3 | 1.9 | 1.8 | 1.0 |
|  | Fisher's exact test (P value) |  | 0.1587 | 0.4490 | 0.0252 | 0.1061 | 0.1510 | 1.0000 |
