# Supplementary material for: Transgenerational Stress Memory Is Not a General Response in Arabidopsis
Source: PLoS One. 2009 Apr 21;4(4):e5202. doi: 10.1371/journal.pone.0005202 (PMC2668180; doi:10.1371/journal.pone.0005202)
Supplement: Table S7 — The effect of radiomimetic (bleocin) stress on the frequency of SHR in the S2 generation (0.06 MB DOC) [file pone.0005202.s009.doc]

**Supplementary Table 7: The effect of radiomimetic (bleocin) stress on the frequency of SHR in the S2 generation**

| Generation |  | S2 | S2 | S2 | S2 | S2 | S2 | S2 |
| --- | --- | --- | --- | --- | --- | --- | --- | --- |
| Pre-growth | Medium | GM | GM | GM | GM | GM | GM | GM |
|  | Day length | 16 h | 16 h | 16 h | 16 h | 16 h | 16 h | 16 h |
|  | Temperature | 22°C | 22°C | 22°C | 22°C | 22°C | 22°C | 22°C |
|  | Duration | 17 d | 17 d | 17 d | 17 d | 17 d | 17 d | 17 d |
|  | Transplanted | no | no | no | no | no | no | no |
| Stress | Treatment | **MOCK bleocin S2** | **10 ng/ml bleocin S2** | **20 ng/ml bleocin S2** | **50 ng/ml bleocin S2** | **100 ng/ml bleocin S2** | **200 ng/ml bleocin S2** | **400 ng/ml bleocin S2** |
|  | Duration of treatment | none | none | none | none | none | none | none |
|  | Recovery | none | none | none | none | none | none | none |
| **11** | Analyzed plants | 60 | 60 | 60 | 60 | 59 | 61 | 58 |
|  | Recombination (GUS spots) | 38 | 64 | 30 | 20 | 35 | 30 | 43 |
|  | GUS spots/plant | 0.633 | 1.067 | 0.500 | 0.333 | 0.593 | 0.492 | 0.741 |
|  | Normalized recombination | 1.000 | 1.684 | 0.789 | 0.526 | 0.937 | 0.777 | 1.171 |
|  | Fold change |  | 1.7 | 0.8 | 0.5 | 0.9 | 0.8 | 1.2 |
|  | Fisher's exact test (P value) |  | 0.0594 | 0.4519 | 0.0555 | 0.8822 | 0.4498 | 0.6654 |
| **1445** | Analyzed plants | 61 | 62 | 62 | 62 | 62 | 62 | 62 |
|  | Recombination (GUS spots) | 24 | 25 | 20 | 21 | 34 | 20 | 19 |
|  | GUS spots/plant | 0.393 | 0.403 | 0.323 | 0.339 | 0.548 | 0.323 | 0.306 |
|  | Normalized recombination | 1.000 | 1.025 | 0.820 | 0.861 | 1.394 | 0.820 | 0.779 |
|  | Fold change |  | 1.0 | 0.8 | 0.9 | 1.4 | 0.8 | 0.8 |
|  | Fisher's exact test (P value) |  | 1.0000 | 0.6020 | 07289 | 0.3402 | 0.6020 | 0.5953 |
