# Supplementary material for: Transgenerational Stress Memory Is Not a General Response in Arabidopsis
Source: PLoS One. 2009 Apr 21;4(4):e5202. doi: 10.1371/journal.pone.0005202 (PMC2668180; doi:10.1371/journal.pone.0005202)
Supplement: Table S8 — The effect of oxidative (paraquat) stress on the frequency of SHR (0.09 MB DOC) [file pone.0005202.s010.doc]

| Generation |  | S0 | S0 | S0 | S0 | S0 | S1 | S1 | S1 | S1 | S1 | S2 | S2 | S2 | S2 | S2 |
| --- | --- | --- | --- | --- | --- | --- | --- | --- | --- | --- | --- | --- | --- | --- | --- | --- |
| Pre-growth | Medium | GM | GM | GM | GM | GM | GM | GM | GM | GM | GM | GM | GM | GM | GM | GM |
|  | Day length | 16 h | 16 h | 16 h | 16 h | 16 h | 16 h | 16 h | 16 h | 16 h | 16 h | 16 h | 16 h | 16 h | 16 h | 16 h |
|  | Temperature | 22°C | 22°C | 22°C | 22°C | 22°C | 22°C | 22°C | 22°C | 22°C | 22°C | 22°C | 22°C | 22°C | 22°C | 22°C |
|  | Duration | 12 d | 12 d | 12 d | 12 d | 12 d | 17 d | 17 d | 17 d | 17 d | 17 d | 17 d | 17 d | 17 d | 17 d | 17 d |
|  | Transplanted | yes | yes | yes | yes | yes | no | no | no | no | no | no | no | no | no | no |
| Stress | Treatment | **MOCK S0** | **0.1 μM paraquat S0** | **0.25 μM paraquat S0** | **0.5 μM paraquat S0** | **1 μM paraquat S0** | **MOCK S1** | **0.1 μM paraquat S1** | **0.25 μM paraquat S1** | **0.5 μM paraquat S1** | **1 μM paraquat S1** | **MOCK S2** | **0.1 μM paraquat S2** | **0.25 μM paraquat S2** | **0.5 μM paraquat S2** | **1 μM paraquat S2** |
|  | Duration of treatment | 5 d | 5 d | 5 d | 5 d | 5 d | none | none | none | none | none | none | none | none | none | none |
|  | Recovery | none | none | none | none | none | none | none | none | none | none | none | none | none | none | none |
| **11** | Analyzed plants | 76 | 72 | 70 | 66 | 71 | 44 | 52 | 55 | 53 | 49 | 56 | 59 | 50 | 54 | 55 |
|  | Recombination (GUS spots) | 81 | 179 | 195 | 76 | 56 | 7 | 53 | 50 | 9 | 9 | 169 | 182 | 217 | 239 | 129 |
|  | GUS spots/plant | 1.066 | 2.486 | 2.786 | 1.152 | 0.789 | 0.159 | 1.019 | 0.909 | 0.170 | 0.184 | 3.018 | 3.085 | 4.340 | 4.426 | 2.345 |
|  | Normalized recombination | 1.000 | 2.333 | 2.614 | 1.080 | 0.740 | 1.000 | 6.407 | 5.714 | 1.067 | 1.155 | 1.000 | 1.022 | 1.438 | 1.467 | 0.777 |
|  | Fold change |  | 2.3 | 2.6 | 1.1 | 0.7 |  | 6.4 | 5.714 | 1.1 | 1.2 |  | 1.0 | 1.4 | 1.5 | 0.8 |
|  | Fisher's exact test (P value) |  | 0.0001 | 0.0001 | 0.8167 | 0.2331 |  | 0.0001 | 0.0001 | 1.0000 | 1.0000 |  | 1.0000 | 0.1003 | 0.0832 | 0.2658 |
| **1445** | Analyzed plants | 60 | 65 | 59 | 59 | 59 | 64 | 55 | 50 |  |  | 52 | 52 | 59 |  |  |
|  | Recombination (GUS spots) | 20 | 17 | 14 | 4 | 3 | 9 | 5 | 3 |  |  | 34 | 34 | 51 |  |  |
|  | GUS spots/plant | 0.333 | 0.262 | 0.237 | 0.068 | 0.051 | 0.141 | 0.091 | 0.060 |  |  | 0.654 | 0.654 | 0.864 |  |  |
|  | Normalized recombination | 1.000 | 0.785 | 0.712 | 0.203 | 0.153 | 1.000 | 0.646 | 0.427 |  |  | 1.000 | 1.000 | 1.322 |  |  |
|  | Fold change |  | 0.8 | 0.7 | 0.2 | 0.2 |  | 0.6 | 0.4 |  |  |  | 1.0 | 1.3 |  |  |
|  | Fisher's exact test (P value) |  | 0.5768 | 0.4394 | 0.0032 | 0.0011 |  | 0.5745 | 0.2380 |  |  |  | 1.0000 | 0.3845 |  |  |

**Supplementary Table 8: The effect of oxidative (paraquat) stress on the frequency of SHR**
