# Supplementary material for: Transgenerational Stress Memory Is Not a General Response in Arabidopsis
Source: PLoS One. 2009 Apr 21;4(4):e5202. doi: 10.1371/journal.pone.0005202 (PMC2668180; doi:10.1371/journal.pone.0005202)
Supplement: Table S9 — The effect of paraquat stress on the frequency of SHR in the progeny of individual S0 plants (0.05 MB DOC) [file pone.0005202.s011.doc]

**Supplementary Table 9: The effect of paraquat stress on the frequency of SHR in the progeny of individual S0 plants**

| Generation |  | S0 | S0 | S1 | S1 | S1 | S1 | S1 | S1 | S1 |
| --- | --- | --- | --- | --- | --- | --- | --- | --- | --- | --- |
| Pre-growth | Medium | GM | GM | GM | GM | GM | GM | GM | GM | GM |
|  | Day length | 16 h | 16 h | 16 h | 16 h | 16 h | 16 h | 16 h | 16 h | 16 h |
|  | Temperature | 22°C | 22°C | 22°C | 22°C | 22°C | 22°C | 22°C | 22°C | 22°C |
|  | Duration | 12 d | 12 d | 12 d | 12 d | 17 d | 17 d | 17 d | 17 d | 17.d |
|  | Transplanted | yes | yes | no | no | no | no | no | no | no |
| Stress | Treatment (**paraquat**) | **MOCK S0** | **0.1 μM paraquat S0** | **MOCK#1 S1** | **MOCK#3 S1** | **0.1 μM Paraquat#1 S1** | **0.1 μM Paraquat#2 S1** | **0.1 μM Paraquat#3 S1** | **0.1 μM Paraquat#4 S1** | **0.1 μM Paraquat#5 S1** |
|  | Duration of treatment | 5 d | 5 d | none | none | none | none | none | none | none |
|  | Recovery | none | none | none | none | none | none | none | none | none |
| **11** | Analyzed plants | 58 | 54 | 29 | 41 | 44 | 50 | 44 | 34 | 34 |
|  | Recombination (GUS spots) | 19 | 39 | 172 | 250 | 155 | 343 | 253 | 208 | 226 |
|  | GUS spots/plant | 0.328 | 0.722 | 5.931 | 6.098 | 3.523 | 6.860 | 5.750 | 6.294 | 6.647 |
|  | Normalized recombination | 1.000 | 2.205 | 1.000 | 1.000 | 0.594 | 1.157 | 0.969 | 1.061 | 1.121 |
|  | Fold change (mean from 3 values) |  | 2.2 |  |  | 0.6 | 1.1 | 1.0 | 1.0 | 1.1 |
|  | Fisher's exact test (P value) |  | 0.02280 |  |  | 0.0130 | 0.5539 | 0.8349 | 1.0000 | 0.7393 |
