# Supplementary material for: Transgenerational Stress Memory Is Not a General Response in Arabidopsis
Source: PLoS One. 2009 Apr 21;4(4):e5202. doi: 10.1371/journal.pone.0005202 (PMC2668180; doi:10.1371/journal.pone.0005202)
Supplement: Table S10 — The effect of UV-B stress on the frequency of SHR (0.06 MB DOC) [file pone.0005202.s012.doc]

**Supplementary Table 10: The effect of UV-B stress on the frequency of SHR**

| Generation |  | S0 | S0 | S0 | S0 | S1 | S1 | S1 | S1 | S2 | S2 | S2 | S2 |
| --- | --- | --- | --- | --- | --- | --- | --- | --- | --- | --- | --- | --- | --- |
| Pre-growth | Medium | PNS | PNS | PNS | PNS | PNS | PNS | PNS | PNS | PNS | PNS | PNS | PNS |
|  | Day length | 12 h | 12 h | 12 h | 12 h | 12 h | 12 h | 12 h | 12 h | 12 h | 12 h | 12 h | 12 h |
|  | Temperature | 22°C | 22°C | 22°C | 22°C | 22°C | 22°C | 22°C | 22°C | 22°C | 22°C | 22°C | 22°C |
|  | Duration | 12 d | 12 d | 12 d | 12 d | 12 d | 12 d | 12 d | 12 d | 12 d | 12 d | 12 d | 12 d |
|  | Transplanted | yes | yes | yes | yes | no | no | no | no | no | no | no | no |
| Stress | Treatment | **mock S0** | **UV-B 3.1 kJ/m^2^/day (1 h) S0** | **UV-B 4.7 kJ/m^2^/day (1.5 h) S0** | **UV-B 6.3 kJ/m^2^/day (2 h) S0** | **mock S0** | **UV-B 3.1 kJ/m^2^/day (1 h) S0** | **UV-B 4.7 kJ/m^2^/day (1.5 h) S0** | **UV-B 6.3 kJ/m^2^/day (2 h) S0** | **mock S0** | **UV-B 3.1 kJ/m^2^/day (1 h) S0** | **UV-B 4.7 kJ/m^2^/day (1.5 h) S0** | **UV-B 6.3 kJ/m^2^/day (2 h) S0** |
|  | Duration of treatment | none | 8 d | 8 d | 8 d | none | none | none | none | none | none | none | none |
|  | Recovery | none | none | none | none | none | none | none | none | none | none | none | none |
| **1445** | Analyzed plants | 46 | 39 | 41 | 37 | 122 | 107 | 102 | 100 | 29 | 29 | 61 | 52 |
|  | Recombination (GUS spots) | 6 | 25 | 39 | 36 | 12 | 11 | 16 | 15 | 2 | 5 | 14 | 12 |
|  | GUS spots/plant | 0.130 | 0.641 | 0.951 | 0.973 | 0.098 | 0.103 | 0.157 | 0.150 | 0.069 | 0.172 | 0.230 | 0.231 |
|  | Normalized recombination | 1.000 | 4.915 | 7.293 | 7.459 | 1.000 | 1.045 | 1.595 | 1.525 | 1.000 | 2.500 | 3.328 | 3.346 |
|  | Fold change |  | 4.9 | 7.3 | 7.4 |  | 1.1 | 1.6 | 1.5 |  | 2.5 | 3.3 | 3.3 |
|  | Fisher's exact test (P value) |  | 0.0013 | 0.0001 | 0.0001 |  | 1.0000 | 0.3156 | 0.3148 |  | 0.4301 | 0.1420 | 0.1350 |
