# Supplementary material for: Transgenerational Stress Memory Is Not a General Response in Arabidopsis
Source: PLoS One. 2009 Apr 21;4(4):e5202. doi: 10.1371/journal.pone.0005202 (PMC2668180; doi:10.1371/journal.pone.0005202)
Supplement: Table S11 — The effect of UV-C stress on the frequency of SHR (0.08 MB DOC) [file pone.0005202.s013.doc]

**Supplementary Table 11: The effect of UV-C stress on the frequency of SHR**

| Generation |  | S0 | S0 | S0 | S0 | S1 | S1 | S1 | S1 | S2 | S2 | S2 | S2 |
| --- | --- | --- | --- | --- | --- | --- | --- | --- | --- | --- | --- | --- | --- |
| Pre-growth | Medium | GM | GM | GM | GM | GM | GM | GM | GM | GM | GM | GM | GM |
|  | Day length | 16 h | 16 h | 16 h | 16 h | 16 h | 16 h | 16 h | 16 h | 16 h | 16 h | 16 h | 16 h |
|  | Temperature | 22°C | 22°C | 22°C | 22°C | 22°C | 22°C | 22°C | 22°C | 22°C | 22°C | 22°C | 22°C |
|  | Duration | 17 d | 17 d | 17 d | 17 d | 17 d | 17 d | 17 d | 17 d | 17 d | 17 d | 17 d | 17 d |
|  | Transplanted | no | no | no | no | no | no | no | no | no | no | no | no |
| Stress | Treatment | **MOCK S0** | **UV-C 1 x 750 J/m^2^ S0** | **UV-C 2 x 1500 J/m^2^ S0** | **UV-C 1 x 3000 J/m^2^ S0** | **MOCK S1** | **UV-C 1 x 750 J/m^2^ S1** | **UV-C 2 x 1500 J/m^2^ S1** | **UV-C 1 x 3000 J/m^2^ S1** | **MOCK S2** | **UV-C 1 x 750 J/m^2^ S2** | **UV-C 2 x 1500 J/m^2^ S2** | **UV-C 1 x 3000 J/m^2^ S2** |
|  | Duration of treatment | none | 10-40 sec (according to dose) | | | none | none | none | none | none | none | none | none |
|  | Recovery | none | 7 d | 7 d | 7 d | none | none | none | none | none | none | none | none |
| **11** | Analyzed plants | 86 | 96 | 74 | 96 |  |  |  |  |  |  |  |  |
|  | Recombination (GUS spots) | 113 | 135 | 61 | 101 |  |  |  |  |  |  |  |  |
|  | GUS spots/plant | 1.314 | 1.406 | 0.824 | 1.052 |  |  |  |  |  |  |  |  |
|  | Normalized recombination | 1.000 | 1.070 | 0.627 | 0.801 |  |  |  |  |  |  |  |  |
|  | Fold change |  | 1.1 | 0.6 | 0.8 |  |  |  |  |  |  |  |  |
|  | Fisher's exact test (P value) |  | 0.7693 | 0.0445 | 0.3172 |  |  |  |  |  |  |  |  |
| **1445** | Analyzed plants | 87 | 100 | 99 | 84 | 156 | 141 | 98 | 132 | 49 | 62 |  | 33 |
|  | Recombination (GUS spots) | 78 | 435 | 569 | 579 | 344 | 238 | 46 | 211 | 163 | 92 |  | 19 |
|  | GUS spots/plant | 0.897 | 4.350 | 5.747 | 6.893 | 2.205 | 1.688 | 0.469 | 1.598 | 1.102 | 1.484 |  | 0.576 |
|  | Normalized recombination | 1.000 | 4.852 | 6.411 | 7.688 | 1.000 | 0.765 | 0.735 | 0.725 | 1.000 | 1.490 |  | 0.792 |
|  | Fold change |  | 4.9 | 6.4 | 7.7 |  | 0.8 | 0.1 | 0.7 | 0.3 | 0.2 |  | 0.1 |
|  | Fisher's exact test (P value) |  | 0.0001 | 0.0001 | 0.0001 |  | 0.0718 | 0.0493 | 0.032 |  | 0.0050 |  | 0.0001 |
