# Supplementary material for: Transgenerational Stress Memory Is Not a General Response in Arabidopsis
Source: PLoS One. 2009 Apr 21;4(4):e5202. doi: 10.1371/journal.pone.0005202 (PMC2668180; doi:10.1371/journal.pone.0005202)
Supplement: Table S12 — The effect of DNA demethylation (zebularine) stress on the frequency of SHR in the S0 generation (0.08 MB DOC) [file pone.0005202.s014.doc]

**Supplementary Table 12: The effect of DNA demethylation (zebularine) stress on the frequency of SHR in the S0 generation**

| Generation |  | S0 | S0 | S0 | S0 |
| --- | --- | --- | --- | --- | --- |
| Pre-growth | Medium | GM | GM | GM | GM |
|  | Day length | 16 h | 16 h | 16 h | 16 h |
|  | Temperature | 22°C | 22°C | 22°C | 22°C |
|  | Duration | 17 d | see stress | see stress | see stress |
|  | Transplanted | yes | yes | yes | yes |
| Stress | Treatment | **MOCK S0** | **20 μM zebularine S0** | **40 μM zebularine S0** | **80 μM zebularine S0** |
|  | Duration of treatment | none | 3 d | 3 d | 3 d |
|  | Recovery | none | 14 d | 14 d | 14 d |
| **651** | Analyzed plants | 73 | 68 | 68 | 68 |
|  | Recombination (GUS spots) | 11 | 387 | 382 | 395 |
|  | GUS spots/plant | 0.151 | 5.691 | 5.618 | 5.809 |
|  | Normalized recombination | 1.000 | 37.769 | 37.281 | 38.549 |
|  | Fold change |  | 37.8 | 37.3 | 38.5 |
|  | Fisher's exact test (P value) |  | 0.0001 | 0.0001 | 0.0001 |
| **11** | Analyzed plants | 76 | 71 | 81 | 65 |
|  | Recombination (GUS spots) | 49 | 307 | 661 | 311 |
|  | GUS spots/plant | 0.645 | 4.324 | 8.160 | 4.785 |
|  | Normalized recombination | 1.000 | 6.707 | 12.657 | 7.421 |
|  | Fold change |  | 6.7 | 12.7 | 7.4 |
|  | Fisher's exact test (P value) |  | 0.0001 | 0.0001 | 0.0001 |
| **IC9** | Analyzed plants | 80 | 82 | 75 | 69 |
|  | Recombination (GUS spots) | 4 | 198 | 170 | 299 |
|  | GUS spots/plant | 0.05 | 2.415 | 2.267 | 4.333 |
|  | Normalized recombination | 1.000 | 48.293 | 45.333 | 86.667 |
|  | Fold change |  | 48.3 | 45.3 | 86.7 |
|  | Fisher's exact test (P value) |  | 0.0001 | 0.0001 | 0.0001 |
| **1445** | Analyzed plants | 69 | 70 | 84 | 68 |
|  | Recombination (GUS spots) | 2 | 154 | 349 | 300 |
|  | GUS spots/plant | 0.029 | 2.200 | 4.155 | 4.412 |
|  | Normalized recombination | 1.000 | 75.900 | 143.339 | 152.206 |
|  | Fold change |  | 75.9 | 143.3 | 152.2 |
|  | Fisher's exact test (P value) |  | 0.0001 | 0.0001 | 0.0001 |
