# Supplementary material for: Transgenerational Stress Memory Is Not a General Response in Arabidopsis
Source: PLoS One. 2009 Apr 21;4(4):e5202. doi: 10.1371/journal.pone.0005202 (PMC2668180; doi:10.1371/journal.pone.0005202)
Supplement: Table S13 — The effect of DNA demethylation (zebularine) stress on the frequency of SHR in the S1 generation (0.10 MB DOC) [file pone.0005202.s015.doc]

**Supplementary Table 13: The effect of DNA demethylation (zebularine) stress on the frequency of SHR in the S1 generation**

| Generation |  | S1 | S0 | S1 | S1 | S1 | S1 | S1 | S1 | S1 |
| --- | --- | --- | --- | --- | --- | --- | --- | --- | --- | --- |
| Pre-growth | Medium | GM | GM | GM | GM | GM | GM | GM | GM | GM |
|  | Day length | 16 h | 16 h | 16 h | 16 h | 16 h | 16 h | 16 h | 16 h | 16 h |
|  | Temperature | 22°C | 22°C | 22°C | 22°C | 22°C | 22°C | 22°C | 22°C | 22°C |
|  | Duration | 21 d | 21 d | 21 d | 21 d | 21 d | 21 d | 21 d | 21 d | 21 d |
|  | Transplanted | no | no | no | no | no | no | no | no | no |
| Stress | Treatment | **MOCK S1** | **MOCK S0 grown in parallel with S1** | **20 μM zebularine S1** | **40 μM zebularine S1** | **80 μM zebularine S1** | **MOCK S1 repeat** | **20 μM zebularine S1 repeat** | **40 μM zebularine S1 repeat** | **80 μM zebularine S1 repeat** |
|  | Duration of treatment | none | none | none | none | none | none | none | none | none |
|  | Recovery | none | none | none | none | none | none | none | none | none |
| **651** | Analyzed plants | 73 | 96 | 86 | 88 | 82 | 100 | 86 | 83 | 102 |
|  | Recombination (GUS spots) | 10 | 115 | 175 | 114 | 130 | 25 | 24 | 21 | 38 |
|  | GUS spots/plant | 0.137 | 1.198 | 2.035 | 1.295 | 1.585 | 0.250 | 0.279 | 0.253 | 0.373 |
|  | Normalized recombination | 1.000 | 8.745 | 14.855 | 9.457 | 11.573 | 1.000 | 1.116 | 1.012 | 1.490 |
|  | Fold change |  | 8.7 | 14.9 | 9.5 | 11.6 |  | 1.1 | 1.0 | 1.5 |
|  | Fisher's exact test (P value) |  |  | 0.0001 | 0.0001 | 0.0001 |  | 0.7500 | 1.0000 | 0.1946 |
| **11** | Analyzed plants | 89 |  | 95 | 97 | 100 |  |  |  |  |
|  | Recombination (GUS spots) | 277 |  | 352 | 559 | 380 |  |  |  |  |
|  | GUS spots/plant | 3.112 |  | 3.705 | 5.763 | 3.800 |  |  |  |  |
|  | Normalized recombination | 1.000 |  | 1.190 | 1.852 | 1.221 |  |  |  |  |
|  | Fold change |  |  | 1.2 | 1.9 | 1.2 |  |  |  |  |
|  | Fisher's exact test (P value) |  |  | 0.3128 | 0.0002 | 0.2439 |  |  |  |  |
| **IC9** | Analyzed plants | 110 |  | 104 | 114 | 85 |  |  |  |  |
|  | Recombination (GUS spots) | 20 |  | 17 | 29 | 13 |  |  |  |  |
|  | GUS spots/plant | 0.182 |  | 0163 | 0.254 | 0.153 |  |  |  |  |
|  | Normalized recombination | 1.000 |  | 0.899 | 1.399 | 0.841 |  |  |  |  |
|  | Fold change |  |  | 0.9 | 1.4 | 0.8 |  |  |  |  |
|  | Fisher's exact test (P value) |  |  | 0.8591 | 0.3443 | 0.7069 |  |  |  |  |
| **1445** | Analyzed plants | 107 |  | 99 | 109 | 101 |  |  |  |  |
|  | Recombination (GUS spots) | 90 |  | 89 | 88 | 59 |  |  |  |  |
|  | GUS spots/plant | 0.841 |  | 0.899 | 0.807 | 0.584 |  |  |  |  |
|  | Normalized recombination | 1.000 |  | 1.069 | 0.960 | 0.694 |  |  |  |  |
|  | Fold change |  |  | 1.1 | 1.0 | 0.7 |  |  |  |  |
|  | Fisher's exact test (P value) |  |  | 0.7600 | 0.9194 | 0.1058 |  |  |  |  |
