# Supplementary material for: Transgenerational Stress Memory Is Not a General Response in Arabidopsis
Source: PLoS One. 2009 Apr 21;4(4):e5202. doi: 10.1371/journal.pone.0005202 (PMC2668180; doi:10.1371/journal.pone.0005202)
Supplement: Table S14 — The effect of DNA demethylation (zebularine) stress on the frequency of SHR in the S2 generation (0.08 MB DOC) [file pone.0005202.s016.doc]

**Supplementary Table 14: The effect of DNA demethylation (zebularine) stress on the frequency of SHR in the S2 generation**

| Generation |  | S2 | S2 | S2 | S2 |
| --- | --- | --- | --- | --- | --- |
| Pre-growth | Medium | GM | GM | GM | GM |
|  | Day length | 16 h | 16 h | 16 h | 16 h |
|  | Temperature | 22°C | 22°C | 22°C | 22°C |
|  | Duration | 21 d | 21 d | 21 d | 21 d |
|  | Transplanted | no | no | no | no |
| Stress | Treatment | **MOCK S2** | **20μM zebularine S2** | **40μM zebularine S2** | **80μM zebularine S2** |
|  | Duration of treatment | none | none | none | none |
|  | Recovery | none | none | none | none |
| **651** | Analyzed plants |  |  |  |  |
|  | Recombination (GUS spots) |  |  |  |  |
|  | GUS spots/plant |  |  |  |  |
|  | Normalized recombination |  |  |  |  |
|  | Fold change |  |  |  |  |
|  | Fisher's exact test (P value) |  |  |  |  |
| **11** | Analyzed plants | 112 | 105 | 110 | 106 |
|  | Recombination (GUS spots) | 370 | 602 | 319 | 385 |
|  | GUS spots/plant | 3.304 | 5.733 | 2.900 | 3.632 |
|  | Normalized recombination | 1.000 | 1.735 | 0.878 | 1.099 |
|  | Fold change |  | 1.7 | 0.9 | 1.1 |
|  | Fisher's exact test (P value) |  | 0.0003 | 0.4395 | 0.5397 |
| **IC9** | Analyzed plants |  |  |  |  |
|  | Recombination (GUS spots) |  |  |  |  |
|  | GUS spots/plant |  |  |  |  |
|  | Normalized recombination |  |  |  |  |
|  | Fold change |  |  |  |  |
|  | Fisher's exact test (P value) |  |  |  |  |
| **1445** | Analyzed plants | 103 | 98 | 121 | 121 |
|  | Recombination (GUS spots) | 366 | 276 | 372 | 261 |
|  | GUS spots/plant | 3.553 | 2.816 | 3.796 | 2.663 |
|  | Normalized recombination | 1.000 | 0.793 | 1.068 | 0.749 |
|  | Fold change |  | 0.8 | 1.1 | 0.7 |
|  | Fisher's exact test (P value) |  | 0.1668 | 0.3603 | 0.0017 |
