# Supplementary material for: Transgenerational Stress Memory Is Not a General Response in Arabidopsis
Source: PLoS One. 2009 Apr 21;4(4):e5202. doi: 10.1371/journal.pone.0005202 (PMC2668180; doi:10.1371/journal.pone.0005202)
Supplement: Table S15 — The effect of histone hyperacetylation by trichostatin A (TSA) stress on the frequency of SHR (0.06 MB DOC) [file pone.0005202.s017.doc]

**Supplementary Table 15: The effect of histone hyperacetylation by trichostatin A (TSA) stress on the frequency of SHR**

| Generation |  | S0 | S0 | S1 | S1 | S2 | S2 |
| --- | --- | --- | --- | --- | --- | --- | --- |
| Pre-growth | Medium | GM | GM | GM | GM | GM | GM |
|  | Day length | 16 h | 16 h | 16 h | 16 h | 16 h | 16 h |
|  | Temperature | 22°C | 22°C | 22°C | 22°C | 22°C | 22°C |
|  | Duration | 12 d | 12 d | 17 d | 17 d | 17 d | 17 d |
|  | Transplanted | yes | yes | no | no | no | no |
| Stress | Treatment | **MOCK S0** | **TSA 1 μg/ml S0** | **MOCK S1** | **TSA 1 μg/ml S1** | **MOCK S2** | **TSA 1 μg/ml S2** |
|  | Duration of treatment | none | germ. on TSA | none | none | none | none |
|  | Recovery | none | none | none | none | none | none |
| **11** | Analyzed plants | 103 | 81 |  |  |  |  |
|  | Recombination (GUS spots) | 344 | 52 |  |  |  |  |
|  | GUS spots/plant | 3.340 | 0.642 |  |  |  |  |
|  | Normalized recombination | 4.622 | 1.646 |  |  |  |  |
|  | Fold change | 0.455 | 0.183 |  |  |  |  |
|  | Fisher's exact test (P value) |  | 0.0000001 |  |  |  |  |
| **1445** | Analyzed plants | 80 | 86 | 56 | 96 | 84 | 84 |
|  | Recombination (GUS spots) | 10 | 28 | 3 | 6 | 3 | 3 |
|  | GUS spots/plant | 0.125 | 0.326 | 0.054 | 0.063 | 0.036 | 0.036 |
|  | Normalized recombination | 1.000 | 2.605 | 1.000 | 1.167 | 1.000 | 1.000 |
|  | Fold change |  | 2.6 |  | 1.2 |  | 1.0 |
|  | Fisher's exact test (P value) |  | 0.0181 |  | 1.0000 |  | 1.0000 |
