# Supplementary material for: Transgenerational Stress Memory Is Not a General Response in Arabidopsis
Source: PLoS One. 2009 Apr 21;4(4):e5202. doi: 10.1371/journal.pone.0005202 (PMC2668180; doi:10.1371/journal.pone.0005202)
Supplement: Table S16 — The effect of histone hyperacetylation (sodium butyrate) stress on the frequency of SHR (0.06 MB DOC) [file pone.0005202.s018.doc]

**Supplementary Table 11: The effect of histone hyperacetylation (sodium butyrate) stress on the frequency of SHR**

| Generation |  | S0 | S0 | S1 | S1 | S2 | S2 |
| --- | --- | --- | --- | --- | --- | --- | --- |
| Pre-growth | Medium | GM | GM | GM | GM | GM | GM |
|  | Day length | 16 h | 16 h | 16 h | 16 h | 16 h | 16 h |
|  | Temperature | 22°C | 22°C | 22°C | 22°C | 22°C | 22°C |
|  | Duration | 12 d | 12 d | 17 d | 17 d | 17 d | 17 d |
|  | Transplanted | yes | yes | no | no | no | no |
| Stress | Treatment | **MOCK S0** | **0.4 mM sodium-butyrate S0** | **MOCK S1** | **0.4 mM sodium-butyrate S1** | **MOCK S2** | **0.4 mM sodium-butyrate S2** |
|  | Duration of treatment | none | germ. on butyrate | none | none | none | none |
|  | Recovery | none | none | none | none | none | none |
| **11** | Analyzed plants | 103 | 75 |  |  |  |  |
|  | Recombination (GUS spots) | 344 | 188 |  |  |  |  |
|  | GUS spots/plant | 3.340 | 2.507 |  |  |  |  |
|  | Normalized recombination | 4.622 | 2.344 |  |  |  |  |
|  | Fold change | 0.455 | 0.271 |  |  |  |  |
|  | Fisher's exact test (P value) |  | 0.0191 |  |  |  |  |
| **1445** | Analyzed plants | 80 | 85 | 56 | 75 | 84 | 73 |
|  | Recombination (GUS spots) | 10 | 50 | 3 | 3 | 3 | 2 |
|  | GUS spots/plant | 0.125 | 0.588 | 0.054 | 0.040 | 0.036 | 0.027 |
|  | Normalized recombination | 1.000 | 4.706 | 1.000 | 0.747 | 1.000 | 0.767 |
|  | Fold change |  | 4.7 |  | 0.7 |  | 0.8 |
|  | Fisher's exact test (P value) |  | 0.0001 |  | 1.000 |  | 1.000 |
