# Supplementary material for: Transgenerational Stress Memory Is Not a General Response in Arabidopsis
Source: PLoS One. 2009 Apr 21;4(4):e5202. doi: 10.1371/journal.pone.0005202 (PMC2668180; doi:10.1371/journal.pone.0005202)
Supplement: Table S17 — The effect of UV-C stress on the frequency of SHR in four different SHR trap lines (0.08 MB DOC) [file pone.0005202.s019.doc]

**Supplementary Table 17: The effect of UV-C stress on the frequency of SHR in four different SHR trap lines**

| Generation |  | S0 | S0 | S0 | S0 |
| --- | --- | --- | --- | --- | --- |
| Pre-growth | Medium | GM | GM | GM | GM |
|  | Day length | 16h | 16h | 16h | 16h |
|  | Temperature | 22°C | 22°C | 22°C | 22°C |
|  | Duration | 17d | 17d | 17d | 17d |
|  | Transplanted | no | no | no | no |
| Stress | Treatment | **MOCK S0** | **UV-C 1 x 1500 J/m2** | **UV-C 1 x 3000 J/m2** | **UV-C 1 x 6000J/m2** |
|  | Duration of treatment | none |  | 20-80 sec (according to dose) |  |
|  | Recovery | none | 5d | 5d | 5d |
| **651** | Analyzed plants | 58 | 69 | 77 | 58 |
|  | Recombinations (GUS spots) | 23 | 31 | 57 | 53 |
|  | GUS spots/plant | 0,397 | 0,449 | 0,740 | 0,914 |
|  | Normalized recombinations | 1,000 | 0,853 | 1,286 | 1,563 |
|  | Fold change |  | 0,9 | 1,3 | 1,6 |
|  | Fisher's exact test (P value) |  | 0,7457 | 0,0422 | 0,0074 |
| **11** | Analyzed plants | 58 | 53 | 66 | 44 |
|  | Recombinations (GUS spots) | 43 | 66 | 28 | 14 |
|  | GUS spots/plant | 0,741 | 1,245 | 0,424 | 0,318 |
|  | Normalized recombinations | 1,000 | 1,680 | 0,341 | 0,750 |
|  | Fold change |  | 1,7 | 0,3 | 0,8 |
|  | Fisher's exact test (P value) |  | 0,0599 | 0,0745 | 0,0253 |
| **IC9** | Analyzed plants | 50 | 51 | 53 | 54 |
|  | Recombinations (GUS spots) | 7 | 31 | 36 | 29 |
|  | GUS spots/plant | 0,140 | 0,608 | 0,679 | 0,537 |
|  | Normalized recombinations | 1,000 | 4,342 | 4,852 | 3,836 |
|  | Fold change |  | 4,3 | 4,9 | 3,8 |
|  | Fisher's exact test (P value) |  | 0,0009 | 0,0003 | 0,003 |
| **1445** | Analyzed plants | 52 | 52 | 52 | 52 |
|  | Recombinations (GUS spots) | 71 | 275 | 273 | 283 |
|  | GUS spots/plant | 1,365 | 5,288 | 5,250 | 5,442 |
|  | Normalized recombinations | 1,000 | 3,873 | 3,845 | 3,986 |
|  | Fold change |  | 3,9 | 3,8 | 4,0 |
|  | Fisher's exact test (P value) |  | 0,0001 | 0,0001 | 0,0001 |
